# Effects of Ultrasonic Dispersion Energy on the Preparation of Amorphous SiO_2_ Nanomaterials for In Vitro Toxicity Testing

**DOI:** 10.3390/nano9010011

**Published:** 2018-12-22

**Authors:** Martin Wiemann, Antje Vennemann, Michael Stintz, Rodrigo R. Retamal Marín, Frank Babick, Gottlieb-Georg Lindner, Tobias B. Schuster, Ulrich Brinkmann, Nils Krueger

**Affiliations:** 1 IBE R&D Institute for Lung Health gGmbH, Mendelstr. 11, D-48149 Münster, Germany; vennemann@ibe-ms.de; 2 Research Group Mechanical Process Engineering, Institute of Process Engineering and Environmental Technology, Technische Universität Dresden, Münchner Platz 3, D-01062 Dresden, Germany; michael.stintz@tu-dresden.de (M.S.); rodrigo.retamal@tu-dresden.de (R.R.R.M.); Frank.Babick@tu-dresden.de (F.B.); 3 Evonik Resource Efficiency GmbH, Brühler Straße 2, 50389 Wesseling, Germany; gottlieb-georg.lindner@evonik.com; 4 Evonik Resource Efficiency GmbH, Rodenbacher Chaussee 4, 63457 Hanau-Wolfgang, Germany; tobias.schuster@evonik.com (T.B.S.); ulrich.brinkmann@evonik.com (U.B.); nils.krueger@evonik.com (N.K.)

**Keywords:** nanomaterials, synthetic amorphous silica, ultrasonic dispersion energy, in vitro testing, NR8383 alveolar macrophage, autophagy, in vitro testing

## Abstract

Synthetic amorphous silica (SAS) constitute a large group of industrial nanomaterials (NM). Based on their different production processes, SAS can be distinguished as precipitated, fumed, gel and colloidal. The biological activity of SAS, e.g., cytotoxicity or inflammatory potential in the lungs is low but has been shown to depend on the particle size, at least for colloidal silica. Therefore, the preparation of suspensions from highly aggregated or agglomerated SAS powder materials is critical. Here we analyzed the influence of ultrasonic dispersion energy on the biologic activity of SAS using NR8383 alveolar macrophage (AM) assay. Fully characterized SAS (7 precipitated, 3 fumed, 3 gel, and 1 colloidal) were dispersed in H_2_O by stirring and filtering through a 5 µm filter. Aqueous suspensions were sonicated with low or high ultrasonic dispersion (USD) energy of 18 or 270 kJ/mL, respectively. A dose range of 11.25–90 µg/mL was administered to the AM under protein-free conditions to detect particle-cell interactions without the attenuating effect of proteins that typically occur in vivo. The release of lactate dehydrogenase (LDH), glucuronidase (GLU), and tumor necrosis factor α (TNF) were measured after 16 h. Hydrogen peroxide (H_2_O_2_) production was assayed after 90 min. The overall pattern of the in vitro response to SAS (12/14) was clearly dose-dependent, except for two SAS which showed very low bioactivity. High USD energy gradually decreased the particle size of precipitated, fumed, and gel SAS whereas the low adverse effect concentrations (LOECs) remained unchanged. Nevertheless, the comparison of dose-response curves revealed slight, but uniform shifts in EC_50_ values (LDH, and partially GLU) for precipitated SAS (6/7), gel SAS (2/3), and fumed SAS (3/3). Release of TNF changed inconsistently with higher ultrasonic dispersion (USD) energy whereas the induction of H_2_O_2_ was diminished in all cases. Electron microscopy and energy dispersive X-ray analysis showed an uptake of SAS into endosomes, lysosomes, endoplasmic reticulum, and different types of phagosomes. The possible effects of different uptake routes are discussed. The study shows that the effect of increased USD energy on the in vitro bioactivity of SAS is surprisingly small. As the in vitro response of AM to different SAS is highly uniform, the production process per se is of minor relevance for toxicity.

## 1. Introduction

Synthetic amorphous silica (SAS) form an important group of industrially relevant nanomaterials (NMs). For example, large quantities of SAS are incorporated in plastics, lacquers and car tires [1]. In consumer products such as cosmetics or food [2,3,4,5] they serve, e.g., as stabilizers, thickeners, or flow enhancing agents [2,4,6,7,8]. The manifold industrial applications of SAS are based upon different physico-chemical properties and different production processes: SAS may be synthesized in an aqueous solution of sodium silicate and this leads to colloidal silica (CS), silica gel (SG), or precipitated silica (PS). SAS is also synthetized in the gaseous phase from SiCl_4_ [9,10], and this material is referred to as pyrogenic or fumed silica (FS). While CS is usually supplied as a stable aqueous suspension of well-dispersed nanoparticles [11], PS, FS and SG are delivered as dry powders which consist of aggregates or agglomerates, both of which are formed by nanosized primary silica (SiO_2_) particles [9,12].

For dry powder nanomaterials especially, inhalation is the main route for a non-intentional uptake of SAS into the body [13,14]. Of note, a considerable number of occupational epidemiology studies have failed to show adverse lung effects in workers with occupational exposure to SAS [15,16,17]. Nevertheless, various types of SAS, including CS, FS, etc. may induce transient lung inflammation in rats following either short-term inhalation [13,18,19] or sub-acute inhalation [9,20,21]. With respect to the 3 R principle, which is to replace, reduce, and refine animal experiments [22], several in vitro studies have investigated the cellular effects of SiO_2_ nanomaterials [2,9,21,23]. Some of these have explicitly studied the possibility of predicting the toxicity potential in vivo [24,25,26].

Recently, an in vitro assay based on the rat alveolar macrophage cell line NR8383 [27,28] was introduced and was shown to predict the toxicity of nanomaterials and non-nanosized materials measured in short-term inhalation studies [29]. It is widely accepted that alveolar macrophages (AMs) are the first line of defence against invading microorganisms and respirable particles in the lung. Also, AMs take up the vast majority of nanoparticles in the lung parenchyma [30,31,32,33]. A severe disturbance or activation of the AM population in the lung will negatively influence lung clearance or have pro-inflammatory effects. Therefore, the relevant in vitro responses to (nano)particles of NR8383 cells include cytotoxicity and activation (release of LDH and GLU), as well as pro-inflammatory effects and oxidative stress (release of TNF and H_2_O_2_). Particles are administered to AM under protein-free conditions, as this allows a more direct particle-cell interaction. Subtle differences in size, composition and structure of particles, which were expected to occur among highly similar SAS qualities, may be discovered this way and lead to differential biological activities. Of note, this approach circumvents the attenuating effects of proteins typically occurring in vivo [29].

However, as for all in vitro assays making use of submerged cells, the dispersion of NMs is a crucial step. This is particularly relevant for the different SAS, which range from the nano-sized particles that are typical for CS, to several hundred micro-meter large aggregates or agglomerates. As outlined by Albers et al. [34], the definitions of these terms are highly relevant for SAS as they differ considerably with respect to agglomerate/aggregate size, and also with respect to included chemical compounds or impurities. According to the EU COM recommendation 696/2011 [21], an aggregate comprises “strongly bound and fused primary particles” whereas an agglomerate is “a collection of weakly bound particles or aggregates, where the resulting external surface area is similar to the sum of the surface areas of the individual components”. A primary particle is defined as the original source particle of aggregates or agglomerates (c.f. [34]).

An in vitro assay designed to determine the particle toxicity within the lung parenchyma should only investigate respirable particles. Therefore, a particle dispersion method is needed that eliminates large aggregates/agglomerates, which would not enter into the deep lung parenchyma. Application of very high ultrasonic dispersion energy of up to 1440 J/mL administered with a sonotrode immersed into the suspension, has been suggested as the method of choice in several recent protocols [35,36,37,38,39,40,41,42,43]. However, although high ultrasonic energy may successfully disintegrate large aggregates, e.g., of PS, the method is time consuming and demands extensive cooling. Most importantly, it bears the risk that metal particles ablated from the tip of the sonotrode during prolonged sonication will contaminate the particle suspension [4]. To circumvent these difficulties, we have previously explored the effects of USD energy on representatives of PS, FS, GS, and CS [4]. We found that administration of 270 J/mL, together with a filtering approach removed aggregates/agglomerates too large for in vitro testing. Furthermore, the effects of increased USD energy on particle size distribution were confined to PS and FS, but were not seen for CS and SG. Therefore, the main hypothesis of the present investigation was that the influence of increased USD energy on the biologic activity of SAS in vitro depends on the SAS production process.

To this aim, we analyzed the outcome of particle dispersion with different USD energies on the NR8383 macrophage assay. Several types of PS (P-1 to P-7), FS (F1 to F-3), GS (G-1 to G-3) and one CS (C-1) were subjected to USD energies of either 270 J/mL or 18 J/mL, which was the standard procedure hitherto used for the macrophage assay. We also analyzed the sedimentation of dispersed SAS in parallel experiments mimicking cell culture conditions. Furthermore, we conducted an electron microscopic study of SAS-exposed NR8383 cells to describe the subcellular distribution of SAS along with indicators of beginning toxicity. Results are presented and discussed in four main chapters, each of which is devoted to the production category of the respective SAS.

## 2. Materials and Methods

### 2.1. Materials

Micron-sized corundum (Elektroschmelzwerk Kempten (ESK), Kempten, Germany) and quartz DQ12 particles (DMT, Essen, Germany) were used as negative and positive particle controls, respectively, and have been characterized in [29]. SAS were provided by Evonik Resource Efficiency GmbH as dry powders except for one colloidal material (C-1), which was delivered as a suspension. Table 1 shows the trade names, and/or abbreviations along with characterization data. Routine methods used for SAS characterization are provided in the footnote of Table 1. Solubility was measured according to OECD Guideline 105, and further specified for the concentration of SAS (50 g SiO_2_/L) and the temperature (20 ± 0.5 °C). Dissolved SiO_2_ was measured by (i) inductively coupled plasma optical emission spectrometry (ICP-OES), and (ii) ultraviolet and visible absorption spectroscopy (UV-VIS) using the molybdate method. To exclude contamination by insufficiently separated fine particulate SiO_2_, a highly sensitive Tyndall device was applied [44]. Solubility values (Table 1) represent the mean of both methods.

### 2.2. Preparation of Particle Suspensions by Ultrasonic Treatment

As the main goal of the study was to compare effects of SAS dispersed with different USD energies on NR8383 cells in vitro, we prepared the samples in such a way that the amount of ultrasonic energy was the only difference between the testing suspensions eventually pipetted onto the cells. To this aim, 50 mg of particles were suspended in 25 mL sterile H_2_O (Aqua ad injectabilia, Braun Melsungen AG, Melsungen, Germany); small differences in the water content of SAS (see Table 1) were neglected. Suspensions were briefly vortexed, and then stirred with a magnetic bar (2 cm) at 700 rpm for 90 min. These suspensions were then passed through a sterile polyamide gauze with a nominal pore width of 5 µm (Bückmann GmbH und Co. KG, Mönchengladbach, Germany) to remove large aggregates/agglomerates (>10 µm) which cannot be engulfed by the cells. Filtration characteristics of the nylon gauze have been analysed before [4]. Particle penetration amounted to 25–100% of the total mass under these conditions and was determined gravimetrically from aliquots of the dried aqueous suspensions. Five materials (P-2, P-3, P-5, P-7, and G-3) were mortared in a porcelain mortar for 5 min to improve permeation of particles through the filter gaze. Filtrates were adjusted to a concentration of 360 µg/mL with sterile double distilled H_2_O (dH_2_O). Five mL of this suspension was then transferred into a 20 mL glass vial and subjected to USD energies of 18 J/mL or 270 J/mL [4]. For this purpose, a 5 mm sonotrode connected to a Branson 450D ultrasonicator (Branson Ultrasonics Corporation, Danbury, CT, USA) was inserted into the suspension with the tip being located approximately 2 mm over the bottom of the glass vial, which was fixed with a clamp-held in an ice-water bath. To achieve the 18 J/mL energy level, the amplitude of the sonotrode was set to 20% and ultrasonic energy was delivered for 12 s. To achieve the 270 J/mL energy level, this treatment was repeated 15 times with a 12 s interval after each round. After each particle treatment, the sonotrode was cleaned in dH_2_O and 70% ethanol. All ultrasonic treatments were carried out with the same sonotrode tip, which showed no signs of ablation. 

### 2.3. Sterility Testing

To test for any fungal or bacterial contaminations, 100 µL of the final suspension as prepared for in vitro testing was plated onto Caso agar and malt extract agar (both from Applichem GmbH, Darmstadt, Germany) and incubated at 37 °C for 3 days. No bacterial or fungal contaminants were detected.

### 2.4. Measurement of Particle Size and Sedimentation

The velocity of gravitational settling (v_sed_) of SAS was derived from analytical centrifugation with a LUMiSizer® 651 (LUM GmbH, Berlin, Germany), which is a cuvette-type photocentrifuge that measures the sedimentation process by spatially resolved turbidity measurements along the radial position using blue light (470 nm) [47,48]. Centrifugation was conducted at 37 °C. The instrument software yielded extinction-weighted velocity distributions (Q(v)/%); their median values were considered as the average settling velocity in the centrifugal field and re-calculated for settling under normal gravity allowing us to obtain v_sed_ (in µm/ s or mm/d). Samples were dispersed in H_2_O with an initial concentration of 2 mg/mL and treated as described (stirring at 700 rpm for 90 min, filtration through 5 µm filter, USD energy set to 18 or 270 J/mL, dilution with double concentrated (2×) F-12K medium). Final concentrations of SAS amounted to approximately 1 mg/mL, which yielded a sufficient scattering signal for analysis, except for C-1, which demanded a higher concentration for detection (5 mg/mL).

Particle size was measured with dynamic light scattering (DLS) using a Nanophox instrument (Sympatec GmbH, Clausthal-Zellerfeld, Germany). DLS signals (i.e., cross-correlation functions) were analyzed with the method of cumulants which provides a characteristic mean particle size (x_cum_, i.e., the harmonic mean of the intensity-weighted size distribution according to DIN ISO 22412:2017), and also the polydispersity index (PDI), which is a dimensionless measure of the distribution width. Samples were put into closed cuvettes (4 mL), which were placed in a temperature-controlled sample holder at least 15 min before the measurements. Under these conditions, the ultrapure H_2_O and F-12K medium were identified as particle-free.

### 2.5. Cultivation of NR8383 Macrophages and Cell Culture Assays

NR8383 cells (ATCC, USA; ATCC^®^ Number: CRL-2192TM) were maintained in F-12K cell culture medium supplemented with 15% fetal calf serum (FCS), 1% penicillin/streptomycin, and 1% L-glutamine (all from PAN Biotech, Aidenbach, Germany) as described in [29]. For the assay, cells were seeded into 96-well plates (3 × 10^5^ cells/well) and kept at 37 °C and 5% CO_2_. Each well contained 200 µL F-12K cell culture medium in which the concentration of FCS was reduced to 5%. After 24 h, the medium was replaced by serum-free test material preparations: to determine the release of LDH, GLU and TNFα from the cells, the test material suspensions were serially diluted to 90, 45, 22.5, and 11.25 µg/mL with serum-free F-12K. To measure release of H_2_O_2_, the same dilutions were prepared in KRPG buffer (129 mM NaCl, 4.86 mM KCl, 1.22 mM CaCl_2_, 15.8 mM NaH_2_PO_4_, 5–10 mM glucose; pH 7.3–7.4). Except for FCS, all reagents were purchased from Sigma-Aldrich (F-12K, Sigma-Aldrich, Taufkirchen, Germany).

Assays were carried out as described in [29]. In brief, H_2_O_2_ released into the supernatant was quantified with the Amplex Red^®^ assay measuring the formation of resorufin (in triplicate). Therefore, optical density was measured photometrically at 570 nm (reference value: 620 nm) with a plate reader (Tecan Infinite F200Pro, Tecan GmbH, Crailsheim, Germany). Measurements were corrected for background absorbance of cell free-particle controls and converted into absolute concentrations of H_2_O_2_ using the molar extinction coefficient of resorufin (54,000 L × mol^−1^ × cm^−1^). LDH activity was measured photometrically (in triplicate) using 50 µL from each well for the Roche Cytotoxicity Kit (Sigma-Aldrich, Taufkirchen, Germany) and measured according to the manufacturer’s protocol. To measure GLU activity, 50 µL of the supernatant (sampled after 16-h test material incubation) were incubated with 100 µL 0.2 M sodium acetate buffer (pH 5) containing 13.3 mM p-nitrophenyl-D-glucuronide and 0.1% Triton X-100. The reaction was terminated after 2 h by addition of 100 µL 0.2 M sodium hydroxide. Optical density was measured at 405 nm. Both the LDH- and GLU-based values were corrected for cell-free adsorption and normalised to the positive control (0.1% Triton X-100 in F-12K) which was set to 100%. Concentration of tumor necrosis factor α (TNF) was determined with a specific enzyme-linked immosorbent assay (ELISA) for rat TNF (Quantikine ELISA Kit) according to the manufacturer’s protocol (Bio-Techne GmbH, Wiesbaden-Nordenstadt, Germany). F-12K assay medium served as vehicle control. As a further control, the TNF-forming capacity of NR8383 cells was confirmed by stimulation with lipopolysaccharide (0.1 µg/mL, Sigma-Aldrich, Taufkirchen, Germany). Notably, aliquots for the determination of LDH, GLU and TNF were taken from the same well.

### 2.6. Particle Size Determination under Cell Culture Conditions with Particle Tracking Analysis 

The particle size distribution in the cell culture medium was determined by parallel particle tracking analyses (PTA) at the end of cell culture testing period. A NanoSight LM10 instrument equipped with a violet laser (405 nm), an Andor CCD camera, and particle tracking software (NTA3.0, Malvern Instruments GmbH, Herrenberg, Germany) was used. Cell-free approaches were carried out using 90 µg/mL of each SAS dispersed with either 18 or 270 J/mL. Suspensions were incubated in H_2_O, KRPG buffer, and F-12K medium under cell culture conditions for 90 min and 16 h, respectively. Suspensions were retrieved from the wells and pipetted onto the laser-stage of the LM-10. Measurable concentrations (approximately 5 × 10^8^ particles/mL) were prepared by serial dilution. Results are presented together with the dilution factor (DF), which may be used to estimate the particle concentration. 

### 2.7. Electron Microscopy of NR8383 Macrophages

To study the subcellular distribution of particles by transmission electron microscopy (TEM) and also by energy dispersive X-ray analysis (TEM-EDX), cells were exposed to selected particle concentrations as described. To avoid excessive cell damage, concentrations of SAS were chosen below the LDH EC50 value. However, to ease embedding and cutting, NR8383 cells were seeded onto small discs (diameter 6 mm) of Melinex embedding film (Plano, Wetzlar, Germany), which had been rinsed in 70% ethanol, dried, and positioned onto the bottom of the wells of microtiter plates. Cells were cultured on these carriers in 200 µL F-12K supplemented with 5% FCS, and allowed to adhere. After one day, the medium was replaced by the SAS-containing serum-free F-12K medium and cells were exposed to the particles under cell culture conditions (100% humidity, 5% CO_2_, 37 °C). After 90 min or 16 h, suspensions were withdrawn and the fixative (2.5% glutardialdehyde in 0.1 M sodium phosphate buffer, pH 7.3) was added for 60 min. Fixed cells were washed three times with the same buffer, post-fixed in 1% OsO_4_, dehydrated in ethanol to the 70% step, and stained en bloc with uranium acetate (1%) for 1 h. Cells were finally dehydrated via ethanol/propylene oxide, and embedded in Epon 812 (Sigma Aldrich, Taufkirchen, Germany). Sections (50–60 nm) were cut perpendicular to the carrier using a diamond knife, and viewed without further staining with a Tecnai G2 electron microscope operated at 120 kV. Images were taken with a Quemesa digital camera (Olympus Soft Imaging Solutions, Münster, Germany). TEM-EDX analyses of selected sections (60–70 nm) was carried out by Evonik Technology & Infrastructure GmbH (Hanau, Germany) using a Jeol 2010F equipped with Pathfinder X-Ray Microanalysis Software (Thermo Scientific).

### 2.8. Statistical Evaluation

In vitro data were generated in triplicate and three independent repetitions were carried out over three consecutive weeks. To test for significant differences, values from each concentration were compared to non-treated controls using 2-way analysis of variance (ANOVA) with Dunnett´s multiple comparisons test. A value of *p* ≤ 0.05 was considered significant (*). All data were expressed as mean ± standard deviation (SD). Calculations of EC50 values and Hill coefficients were carried out with GraphPad Prism software.

## 3. Results and Discussion

### 3.1. Particle Characterization

**Figure 1:**
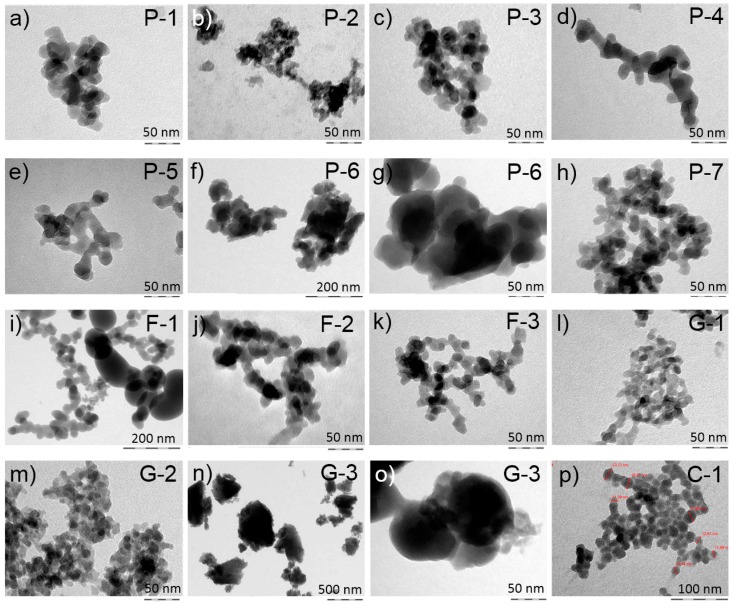
Electron microscopic images of the synthetic amorphous silica (SAS) used in the study. Typical aggregates are shown for precipitated (**a**–**h**), fumed (**i**–**k**), gel (**l**–**o**) and colloidal SAS (**p**). Abbreviations in the upper right corners refer to Table 1.

A total of 14 different SAS were included in the study, comprising 7 precipitated silica (PS), 3 fumed silica (FS), 3 silica gels (SG), and 1 colloidal silica (CS). To obtain a representative selection, we selected materials with very different physico-chemical properties with respect to shape, size and surface properties (Table 1). All materials consisted of nano-sized primary particles whose size, by TEM, ranged from 3.1 to 41.4 nm (Table 1). Aggregates exhibited shapes and complexities typical of the different production processes (Figure 1). The loss on drying was typically low for FS (≤0.6%), between 1.5 and 5 for SG, and 3.8 to 6.5 for PS. 

Specific surface areas, as determined for dried powders and the lyophilized colloidal SAS C-1 by N_2_ adsorption (see Table 1), ranged from 40 m^2^/g (P-6) to 720 m^2^/g (G-1). Adsorption of cetyltrimethylammonium bromide (CTAB), which is a standard method to measure the surface area of precipitated hydrated silica, resulted in highly similar values, except for one, SG (G-1). Sears number, which provides a measure for accessible SiO_2_ groups, ranged from 5 to 16.3 mL/1.5 g. Adsorption of diethylhexyladipate (DOA numbers), which indicates the liquid absorption capacity of silica and depends on pore volume, moisture content, and particle size, ranged from 84 to 349 mL/100 g. 

Zeta potentials in H_2_O were negative for all materials and ranged from −21 mV (P-2) to −70 mV (P-4). As pH values at zero charge were ≤2.8 (C-1: pH 4.4) all materials were negatively charged at pH 7.4 which is the pH value under culture conditions.

Solubility under steady state conditions, as measured by ICP-MS and UV/VIS absorbance, ranged from 89.4 mg/L (P-4) to 226 mg/mL (F-3). Possible relationships between the physico-chemical data and in vitro findings will be discussed below.

### 3.2. In Vitro Toxicity Determination of SAS

The aim of the study was to compare the effects of 18 J/mL and 270 J/mL, of which 18 J/mL is the standard USD energy employed in macrophage assay. Values retrieved from the macrophage model (Table 2) were obtained side-by-side, i.e., particles prepared from the same filtrate were subjected to either 18 or 270 J/mL and tested on the same plate, to minimize intra- and inter-assay variation. Three parameters, namely lactate dehydrogenase (LDH), glucuronidase (GLU) and tumor necrosis factor α (TNF) were measured in the cell culture supernatant after 16 h, whereas the released and accumulated H_2_O_2_ was measured in KRPG buffer 90 min post exposure. Micron-sized corundum and quartz particles were included as negative and positive particle controls, respectively. Measured values are shown in Table 2 and further evaluated in a comparative manner as shown in Table 3.

In general, and with respect to the USD energy applied under standard conditions, the majority of SAS exhibited a very similar pattern of responses characterized by largely uniform cytotoxic effects (LDH) accompanied by a release of GLU, with LOECs ≤22.5 µg/mL for both LDH and GLU. Induction of TNF was mostly biphasic with a maximum between 22.5 and 45 µg/mL and LOECs distributed over the whole concentration range. Release of H_2_O_2_ upon administration of SAS was generally moderate and LOECs were mostly obtained at higher concentrations (≥45 µg/mL). All values exceeded those of the non-treated cell control (Table 3). Apart from these general findings, P-6 and G-3 exhibited a far lower or even no (G-1) detectable bioactivity. Overall, SAS exhibited a relatively high bioactivity in the macrophage assay, at least if mass-per-volume was used as a dose metric.

#### 3.2.1. Precipitated SAS (PS)

##### In Vitro Test with NR8383 Macrophages

**Figure 2:**
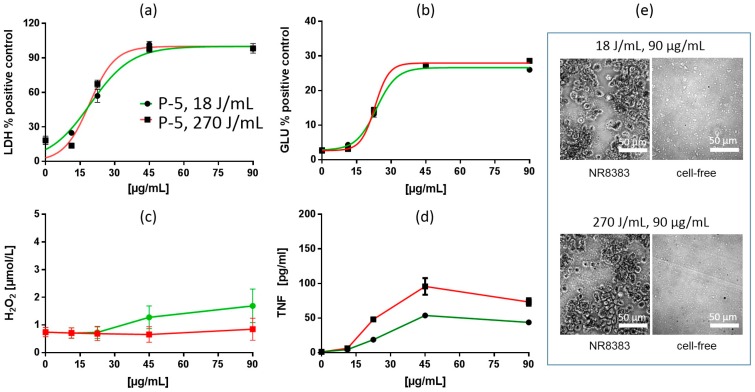
In vitro response of NR8383 alveolar macrophages to precipitated silica P-5. Particles were dispersed with either 18 J/mL (green) or 270 J/mL (red). (**a**) lactate dehydrogenase activity (LDH), (**b**) glucuronidase activity (GLU), (**c**) H_2_O_2_ concentration, and (**d**) tumor necrosis factor alpha (TNF). (**e**) NR8383 cells after 16 h exposure to P-5, dispersed with 18 J/mL or 270 J/mL; right panels show particles settled onto the bottom of culture well under cell-free conditions. P-5-treated cells appear deteriorated and particles are visible between cells were. Note that settled P-5 particles dispersed with 270 J/mL appear smaller than particles dispersed with 18 J/mL.

Treatment of PS with increased USD (270 J/mL) slightly changed the dose-response curves for LDH and GLU, as exemplarily shown for P-5 in Figure 2. Also, LDH curves of P-1, P-2, P-3, P-4, P-5, and P-7 showed an increased slope with Hill coefficients being doubled in some cases (Table 3). EC50 values were slightly diminished by 1.8–10.1% although there was no leftward shift of the dose-response curves. Release of GLU had a shallower dose-response curve than LDH, but slopes were also slightly increased (P-1, P-2, P-3, P-4, P-5, and P-6), as shown in Table 3. Maximum TNF values (see Table 2) were increased in P-1, P-2, P-3, P-4, P-5, P-7, and most LOECS were diminished (Table 3). In contrast, treatment of precipitated SAS with increased USD energy uniformly diminished the dose-dependent release of H_2_O_2_. Taken together, the most typical finding for precipitated SAS was that increased USD energy gradually facilitated the release of LDH, GLU, and TNF, but attenuated the H_2_O_2_ response. 

**Figure 3:**
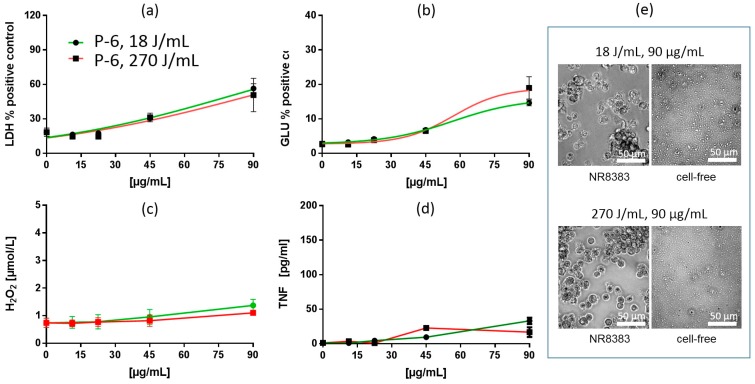
In vitro response of NR8383 alveolar macrophages to precipitated silica P-6. Particles were dispersed with either 18 J/mL (green) or 270 J/mL (red). (**a**) lactate dehydrogenase activity (LDH), (**b**) glucuronidase activity (GLU), (**c**) H_2_O_2_ concentration, and (**d**) tumor necrosis factor alpha (TNF). (**e**) NR8383 cells after 16 h exposure to P-6, dispersed with 18 J/mL or 270 J/mL; right panels show particles settled onto the bottom of culture well under cell-free conditions. P-6-treated cells appear dark due to engulfed particles which had been cleared from the area between the cells. Settled P-6 particles dispersed with 270 J/mL appear smaller than particles dispersed with 18 J/mL.

Figure 3 shows the response to P-6, which had the largest particle size and smallest BET surface of all SAS in this study. Compared to P-5, the effects of P-6 were moderate and hardly modified by the different ultrasonic dispersion energies. Cells appeared healthy after 16 h and had completely cleared particles from the bottom.

##### Particokinetics and Interpretation of In Vitro Findings

As previously shown, precipitated SAS are prone to disintegration by increased USD (270 J/mL) [4], a finding which was visible by phase contrast microscopy for all PS (Figure 2e and Figure 3e). To describe particle size and sedimentation more quantitatively, we performed centrifugation and DLS measurements. For these experiments, we chose P-1 and P-2 as typical representatives of PS. Increased USD (270 J/mL) lowered the particle size (x_cum_) in F12K medium by 59% (P-1) and 28% (P-2), respectively (Table 4). The polydispersity index (PDI) was reduced as well, indicating a narrowed particle size distribution. The rate of sedimentation (v_sed_), which was clearly above 9 mm/d after standard dispersion with 18 J/mL, was reduced upon 270 J/mL by 70.3% (P-1) and 49% (P-2) (Table 4). Given that a v_sed_ of 9 mm/d is sufficient for complete sedimentation of particles in the macrophage assay (filling height: 6 mm, 16 h), the data suggest that the sedimentation of P-2 was complete, whereas that of P-1 was incomplete after increased USD energy (270 J/mL).

The nano-sized particle fraction remaining in F-12K medium (and also in H_2_O) after 16 h under cell culture conditions was studied by PTA. As shown in Table 5, the particle size range of P-1 to P-7 in F-12K medium dropped slightly from 79.9–139.6 (18 J/mL) to 73.9–122 nm (270 J/mL), evidenced also by a leftward shift of the size distribution curves, in both H_2_O and F-12K (Figure S1). Curve maxima were increased (Figure S1), although they were obtained at higher dilution (Table 5). This shows that the number concentration of particles ≤300 nm was higher, if samples were dispersed with 270 J/mL instead of 18 J/mL. However, no further attempts were made to exactly quantify the nano-sized PS particles due to some limitations of the PTA (c.f. [49]). As a whole, increasing USD from 18 to 270 J/mL reduced the particle size of precipitated SAS. This led to smaller particles/agglomerates, delayed particle sedimentation, and increased numbers of nano-sized PS in the medium.

The diminished release of H_2_O_2_ over the whole concentration range (Table 2, Figure 2 and Figure 3) is in line with the lowered sedimentation and accessibility of the larger particles during the 90 min test period. Effects observed after 16 h may be, however, more complex. Thus, the cytotoxic effects of PS dispersed with either 18 J/mL 270 J/mL showed only minor differences, consisting in slightly increased slopes of the dose-response curves of LDH and GLU upon 270 J/mL (especially of P-1 to P-5). Smaller particles are less efficient in reaching the cells by gravitational settling, and this may explain the lowered cytotoxicity (LDH, GLU) of PS dispersed with 270 J/mL in the low concentration range. However, cytotoxicity appeared augmented in the high concentration range (which was different from the rightward shifted dose-response curves of FS, see below). It may be speculated that precipitated SAS re-agglomerate and settle more effectively at higher concentrations under cell culture conditions, and/or that smaller PS are more cytotoxic, as observed for colloidal SAS [50]. Also, the more pronounced formation of TNF in the mid concentration range could be due to increased numbers of smaller particles. In a previous study on four CS, which differed by size (9 nm, 15, 30, and 55 nm) and BET surface (300, 200, 100, 50 m^2^/g), smaller particles elicited progressively more TNF [50]. As the gravitational settling of these particles was low, the effects on cells were assumed to be predominantly caused by diffusing nanoparticles. It is noteworthy that P-6, which has the smallest BET surface and an overall low cytotoxicity, also evoked a very small TNF response. Interestingly, this was also the case for P-4, which has an intermediate BET surface (170 m^2^/g) but the most negative zeta potential (−70 mV), which may prevent particle binding to and/or internalization by the cell. However, more specific experiments are needed to unravel the parameters underlying TNF induction. As a whole, the increased slopes of LDH and GLU curves, as well as changes in TNF induction elicited by PS treated with increased USD energy, may result from a superposition of lowered sedimentation and higher biologic activity of the smaller sub-fraction of PS.

##### Electron Microscopy

While P-1, P-2, P-3, P-5, P-6 and P-7 showed a highly similar cytotoxicity and similar changes if particles were dispersed with higher energy, P-6 elicited a very moderate cytotoxicity under both conditions. To gain insight into underlying mechanisms and differences, we investigated ultrastructural changes of NR8383 alveolar macrophages which had been exposed to either P-6 (67.5 µg/mL) or P-5 (17 µg/mL). For this and all other TEM studies, we chose a single concentration below the respective LDH EC50 value to observe different stages of particle adhesion and uptake in mainly, though not exclusively, intact cells. All TEM studies were confined to SAS dispersed with 18 J/mL because, especially in the sub-EC50 concentration range, differences in toxicity were very small, and therefore, differences in the fine structure of both groups were expected to be hardly discernable.

**Figure 4:**
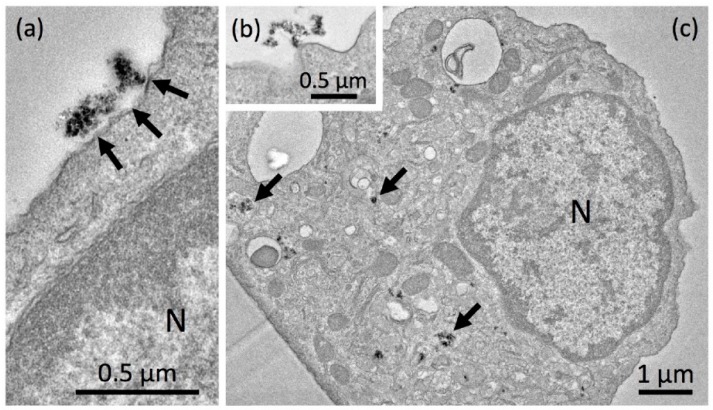
Detection of precipitated P-5 silica nanoparticles in NR8383 cells by transmission electron microscopic (TEM). Cell were treated with 17 µg P-5 per mL in serum-free F-12K and fixed after 90 min. (**a**) P-5 aggregate/agglomerate attached to the cell; the underlying cell membrane appears intact. (**b**) P-5 aggregate/agglomerate close to the site of endosome formation. (**c**) NR8383 macrophage filled with numerous P-5-laden lysosomes and phagolysosomes (arrows).

Figure 4 shows P-5 aggregates/agglomerates attached to the cell membrane (a), and in close vicinity of a forming endosome 90 min after particle administration (b). Although cells were fixed without washing, particles attached to the outer membrane were rarely found (Figure 4c). After 90 min, electron dense nano-sized particle aggregates/agglomerates occurred mainly in lysosomes and phagolysosomes (Figure 4c) or in small vesicles close to electron lucent vacuoles. These observations suggest that P-5 is mainly taken up via small endosomes and further transferred to lysosomes. We found no indication of general membrane damage; also, the cell membrane underneath contacting aggregates/agglomerates appeared intact.

**Figure 5:**
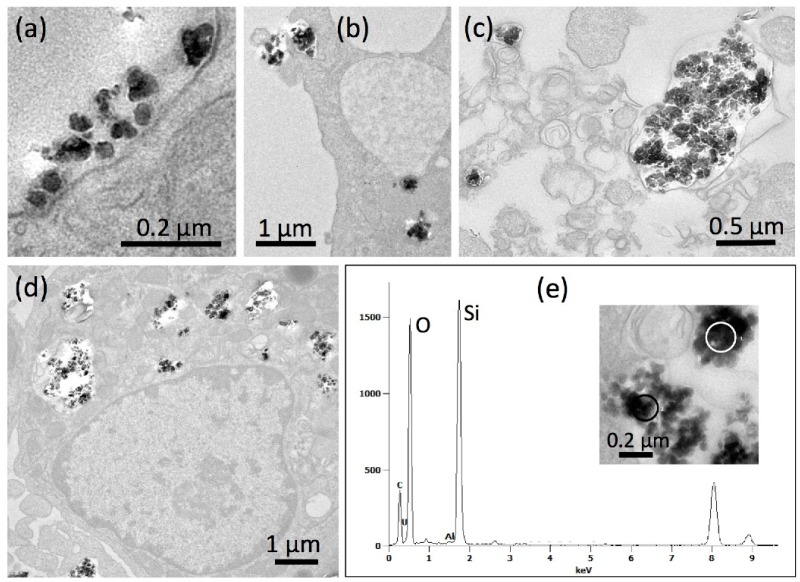
Detection of precipitated P-6 silica nanoparticles in NR8383 cells by transmission electron microscopy (TEM). Cells were treated with 67.5 µg P-6 per mL in serum-free F-12K and fixed after 90 min (**a**,**b**) and 16 h (**c**–**e**). (**a**) P-6 NP attached to the cell; the underlying cell membrane appears undamaged. (**b**) Early stage of phagosome formation (upper left). (**c**) A P-6-filled phagolysosome released from a deteriorated cell. (**d**) NR8383 macrophage filled with numerous P-6-laden phagosomes. (**e**) Energy dispersive X-ray analysis (TEM-EDX) of a P-6-containing phagosome; white circle (inset) marks the analyzed area; results from black circle were highly similar (not shown). Prominent signals (in arbitrary units) were obtained for silicon (Si) and oxygen (O) at typical positions (in keV) of the spectrum.

Aggregates/agglomerates of the less bioactive P-6 particles were also found adhering to the cell membrane (Figure 5a). Again, no indications of outer membrane damage were obtained despite the comparatively high concentration of P-6 (67.5 µg/mL). The primary uptake of P-6 appeared to be mainly, though not exclusively, via larger phagosomes (Figure 5b). Phagocytosis is primarily known as the uptake route for micron-sized particles into alveolar macrophages [51,52]. Uptake of smaller nanoparticles depends on the quality of protein coatings and also on the differentiation state of macrophages [53]. Larger aggregates/agglomerates of P-6 occurred outside the cells (Figure 5c) and often appeared to be taken up as a whole (not shown). After 16 h, cells typically contained numerous large electron lucent phagosomes, which were partly filled with electron dense P-6 particles (Figure 5d). TEM-EDX investigations confirmed that these structures contained prominent amounts of silicon and oxygen (Figure 5e), as expected for SiO_2_. As a whole, P-6 is a more aggregated/agglomerated SAS with a low BET surface, which frequently occurred in phagosomes, and to a lower extent in lysosomes. This is in contrast to P-4 and may be a reason for the comparatively low cytotoxicity (c.f. Figure 3).

#### 3.2.2. Fumed Silica (FS)

##### In Vitro Test with NR8383 Macrophages

**Figure 6:**
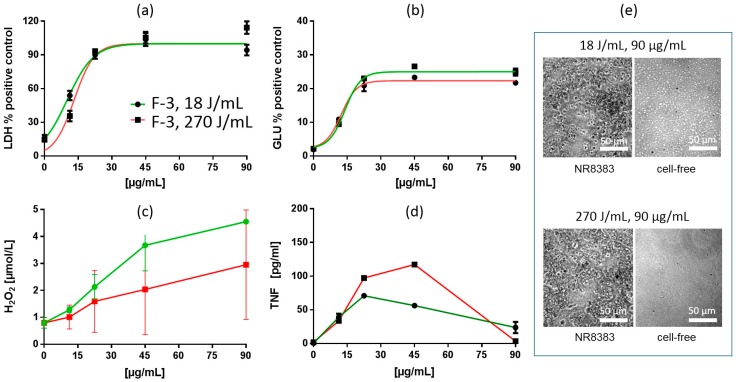
In vitro response of NR8383 alveolar macrophages to fumed silica F-3. Particles were dispersed with either 18 J/mL (green) or 270 J/mL (red). (**a**) lactate dehydrogenase activity (LDH), (**b**) glucuronidase activity (GLU), (**c**) H2O2 concentration, and (**d**) tumor necrosis factor alpha (TNF). (**e**) NR8383 cells after 16 h exposure to P-5, dispersed with 18 J/mL or 270 J/mL; right panels show particles settled onto the bottom of culture well under cell-free conditions. Note that F-3 granules appear smaller at 270 J/mL and that cells treated with both qualities appeared deteriorated.

Treatment of fumed silica suspensions F-1, F-2 and F-3 with increased USD energy (270 J/mL) also changed the dose-response curves for LDH and GLU. However, in contrast to precipitated SAS, LDH and GLU EC50 values were increased by 17.3–39.4% and 13.4–25.1%, respectively. In line with these changes, LDH curves showed a rightward shift (F-1) and/or had an increased slope (F-3) in the low concentration range. GLU curves were also shifted to the right but maximum plateau values were lowered in all cases (F-1, F-2, F-3). Maximum TNF responses were increased (F-1, F-2, F-3), although LOECs remained unchanged (Table 2 and Table 6). Formation of H_2_O_2_ was lowered in all three cases. Thus, the overall finding for fumed SAS was that - with respect to EC50 values - increased USD energy attenuated the release of LDH, GLU, and H_2_O_2_ and allowed increased formation/release of TNF. These changes are exemplarily shown for F-3 (Figure 6).

##### Particokinetics and Interpretation of In Vitro Findings

In general, particles of FS were smaller than PS and SG and exhibited a comparatively narrow size distribution in all media, as reflected by x_cum_ and PDI values, respectively (Table 4). Nevertheless, FS showed a further reduction in particle size upon increased USD energy under cell culture conditions, which was visible by phase contrast microscopy (Figure 6e). DLS measurements showed that 270 J/mL lowered the particle size (x_cum_) of F-1 and F-2 in F-12K medium by 36.6 and 28%, respectively (Table 4). Size reduction was accompanied by a slight reduction of PDI, indicating a narrowed particle size distribution. The calculated rate of sedimentation (v_sed_) amounted to 4.3 (F-1) and 0.6 mm/d (F-2), respectively, and was further reduced by 48.5% (F-1) and 44.3% (F-2) upon 270 J/mL (Table 4).

All sedimentation values were far below 9 mm/d (Table 4), which would—at least in theory—be necessary for complete sedimentation of all particles in a cell culture well (filling height: 6 mm) during the 16 h exposure period. Thus, the sedimentation of FS was incomplete at 18 J/mL and became even lower at 270 J/mL. On the other hand, the nano-sized particle fraction, which hardly settled during 16 h, was increased. Tracking analyses revealed that the particle sizes in F-12K medium (mode values), which ranged from 141 to 154 nm after 16 h (USD: 18 J/mL), dropped by 11.4–22% upon 270 J/mL (Table 5). Therefore, the reduction in particle size of fumed SAS by increasing USD from 18 to 270 J/mL apparently lowered the cellular dose of gravitationally settled particles, and increased the number of small SAS, whose access to cells is diffusion limited.

As observed for PS, changes in the biologic effects of FS dispersed with increased USD energy were relatively low. Smaller particles reach the cells less efficiently by gravitational settling, and this may explain the reduced cytotoxicity (LDH) and H_2_O_2_ responses of FS treated with 270 J/mL. However, FS showed a more pronounced formation of TNF than most PS, as all LOECs were ≤11.25 µg/mL (Table 6). It should be pointed out that FS comprised the highest numbers of small particles in supernatants (Table 5, Figure S1), which is in line with the hypothesis that TNF release is induced by diffusible, slowly settling particles. On the other hand, the reduction of v_sed_ nearly by half as observed for F-1 and F-2 (Table 4), should have shifted the LDH dose-response curves to the right by nearly one doubling step, if the effects were attributable to settled particles. Instead, there was either a very limited shift (F-1), a shift in the low concentration range together with an increased slope at mid concentrations (F-3, Figure 6), or nearly no change (F-2), suggesting that settling FS particles make a minor contribution to the shape of the LDH dose response curves and that further mechanisms are involved as well.

Irrespective of this inhomogeneity, increased USD energy lowered the maximum plateau values of the GLU activity, suggesting an interference of FS (F-1 to F-3) with the enzyme (see also General Discussion below). Interestingly, this reduction was only observed for fumed SAS which are believed to be more hydrophobic and cytotoxic than other SAS modifications [21]. The mechanism underlying the reduction of maximum GLU activity certainly deserves further investigations.

##### Electron Microscopy

**Figure 7:**
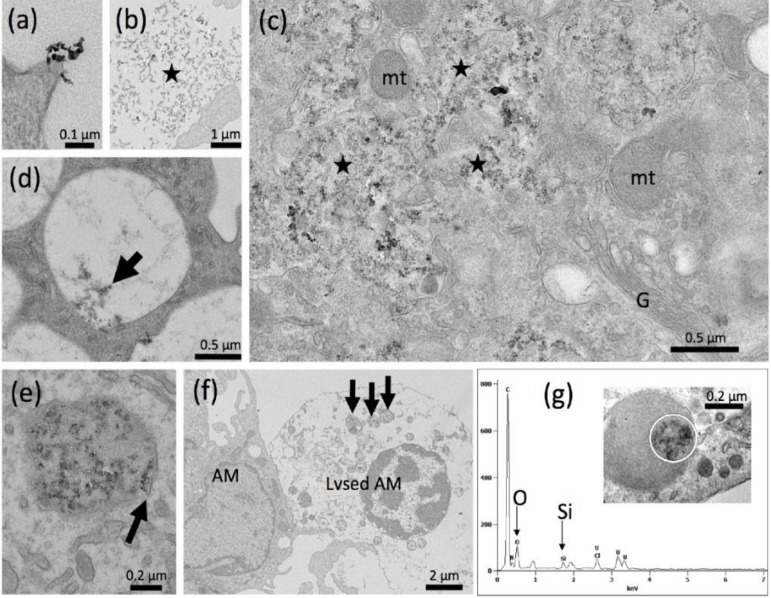
Detection of fumed silica nanoparticles (F-3) in NR8383 cells by transmission electron microscopy (TEM). Cells were treated with 11.25 µg F-3 per mL in serum-free F-12K and fixed after 90 min (**a**–**d**) and after 16 h (**e**,**f**). (**a**) F-3 aggregate/agglomerate attached to a cell. (**b**) Assembly of F-3 particles (asterisk) outside a cell. (**c**) Numerous F-3 particles gathered in endosomal structures of the cytoplasm (asterisks); in the upper right corner electron dense material is enclosed in an autophagosome-like vesicle close to the Golgi apparatus (G) and several mitochondria (mt). (**d**) F-3 particle in a large clear vacuole, and (**e**) within a lysosome. (**f**) A lysed alveolar macrophage (AM) with F-3-laden phagolysosomes (arrows). (**g**) Energy dispersive x-ray analysis (TEM-EDX) of a F-3-laden lysosome (inset), white circle marks analyzed area. Signals (in arbitrary units) were obtained for silicon (Si) and oxygen (O) at typical positions of the spectrum (in keV), confirming that the electron dense material is SiO_2_.

NR8383 alveolar macrophages laden with F-3 (11.25 µg/mL) were investigated by TEM (Figure 7a–g). Electron dense structures were mostly found in lysosomes where their chemical composition was confirmed by TEM-EDX (Figure 7e). However, signals from Si and O were much weaker than observed above for e.g., P-5, suggesting a lower mass of particles gathered within lysosomes. Small aggregates/agglomerates of F-3 were also found attached to the intact outer cell membrane (Figure 7a,b). Interestingly, numerous nano-sized F-3 particles were gathered in tube-like structures resembling the endoplasmic reticulum although no clear membrane boundaries were visible (Figure 7c). Also, autophagosome-like structures contained electron dense material (Figure 7c, upper right). Particulate material was also found in larger clear vesicles (Figure 7d) and lysosomes, some of which showed membrane discontinuities (Figure 7e) suggesting membrane damage of the lytic compartment. F-3 containing phagolysosomes in lysed cells (Figure 7f) suggest that cell lysis has occurred secondary to particle uptake. Overall, F-3 particles did not damage the outer cell membrane but their uptake seemed to compromise the membrane enclosure of lysosomes or of the endoplasmic reticulum, eventually fostering autophagic processes.

#### 3.2.3. Silica Gels (SG)

##### In Vitro Test with NR8383 Macrophages

**Figure 8:**
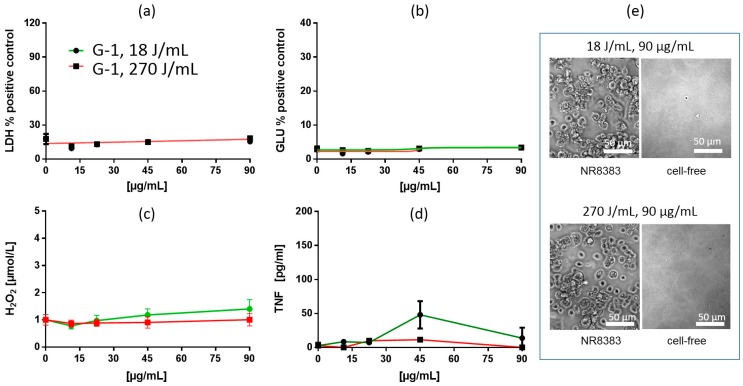
In vitro response of NR8383 alveolar macrophages to silica gel G-3. Particles were dispersed with either 18 J/mL (green) or 270 J/mL (red). (**a**) lactate dehydrogenase activity (LDH), (**b**) glucuronidase activity (GLU), (**c**) H_2_O_2_ concentration, and (**d**) tumor necrosis factor alpha (TNF). (**e**) NR8383 cells after 16 h exposure to P-5, dispersed with 18 J/mL or 270 J/mL; right panels show particles settled onto the bottom of culture well under cell-free conditions. Numerous particles are visible under cell-free conditions and appear similar sized at 18 or 270 J/mL.

Silica gels are compact materials that can hardly be disintegrated by enhanced USD energy [4]. G-2 and G-3 elicited largely similar effects, which were comparable to those of PS. However, G-1 had no apparent effect at all up to the maximum concentration of 90 µg/mL (Table 2 and Table 6). As expected, treatment of G-2 and G-3 with increased USD energy (270 J/mL) induced almost no changes in cytotoxicity, such that dose-response curves EC50 values for LDH were nearly identical (Table 6, Figure 8). The dose-response curves of GLU were shifted slightly leftwards (G-2) or became steeper. The formation of H_2_O_2_ was diminished (G-2 and G-3), whereas the release of TNF was increased (G-2) or remained unchanged (G-3). Considering the missing effects of G-1, the overall effects of SG on NR8383 macrophages appeared heterogeneous.

##### Particokinetics and Interpretation of In Vitro Findings

Since most of the particles were in the low micrometer range under experimental conditions and remained that size after application of increased USD (270 J/mL) (Table 4, Figure 8e), sedimentation velocity was high for G1 (v_sed_ > 100 mm/d for H_2_O and F-12k medium). Similarly, G-2 particles settled quickly and completely (v_sed_: 25–27 mm/min for H_2_O and F-12k medium) due to a mean particle size of 0.65 µm, which dropped slightly to 0.5 µm upon 270 J/mL (Table 4). Phase contrast microscopy confirmed these data and revealed numerous G-3 particles at the bottom of the culture well whose number and size were similar after 18 J/mL and 270 J/mL (Figure 8e). Thus, the major portion of the particle mass, which eventually contributes to the internalized cellular dose, was in the micron-size range. In addition to this particle fraction, PTA revealed a minor sub-micron fraction (<300 nm, see Table 5, Figure S1) measurable only in undiluted (G-1) or 2-fold diluted F-12K medium (G-2). A higher quantity of small particles was found for G-3 by PTA (dilution factor: 16- and 32-fold). Higher USD energy (270 J/mL) slightly decreased the mean particle size of the micron-sized fraction and conversely increased the sub-micron fraction in F-12K medium, confirming our previous results on G-1 [4]. 

Due to the inhomogeneous responses of NR8383 macrophages to G-1, G-2, and G-3, a general conclusion on the biological effects of SG cannot be drawn, although G-2 and G-3 appear to be more representative for SG. Of note, the largely congruent LDH dose-response curves evoked with SG dispersed with either 18 or 270 J/mL is perfectly in line with the reported rigidity of SG against high USD energy [4]. Nevertheless, PTA revealed a 17.3 ± 4.0% reduction in size of the smaller particles upon 270 J/mL under cell culture conditions (mean value from G1 to G-3, see Table 5), and this reduction may account for the small differences seen in GLU, H_2_O_2_ and TNF values.

The low biologic activity of G-1 was unexpected and may be linked to a high degree of compactness and rigidity; although this material showed the highest N_2_ absorption (BET surface: 720 m^2^/g), its CTAB binding was far lower (170 m^2^/g). CTAB, in contrast to N_2_, permeates into larger pores only (approximately 2 nm or larger), thus, the binding of this molecule may reflect the access of larger biomolecules to the surface of SAS.

##### Electron Microscopy

**Figure 9:**
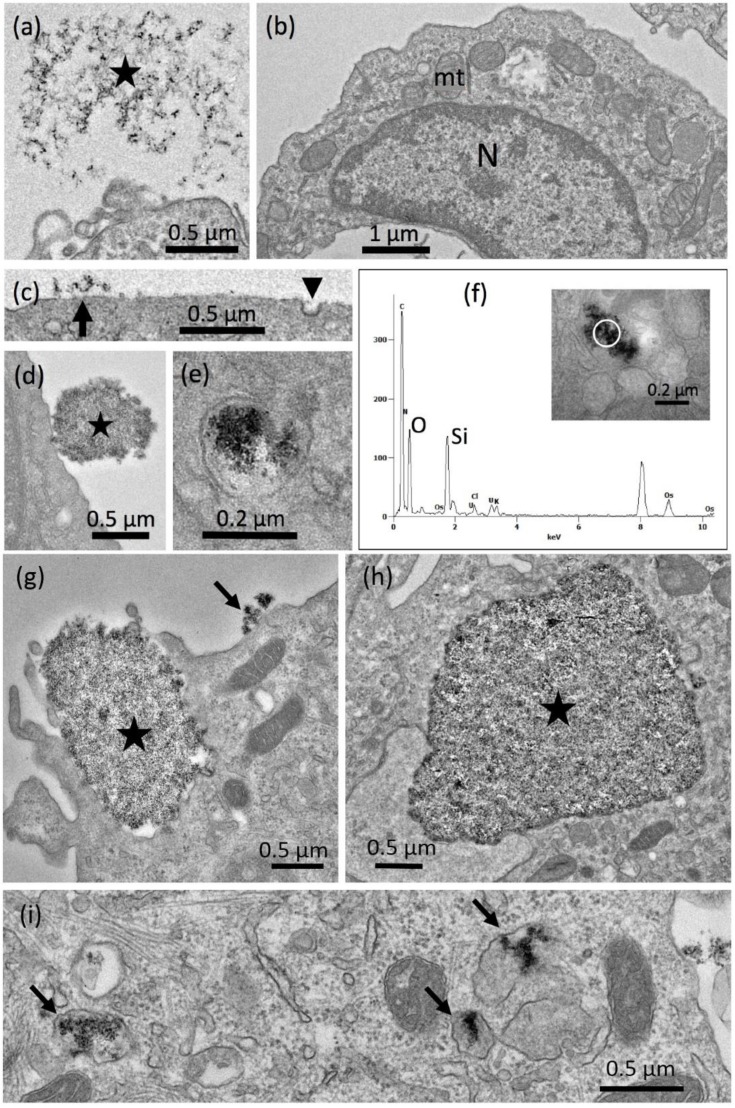
Detection of silica gel nanoparticles in NR8383 cells by transmission electron microscopy (TEM). Cells were treated with 90 µg/mL G-1 (**a**–**f**), or 17 µg/mL G-3 (**g**–**i**) in serum-free F-12K medium and fixed after 90 min (**a**–**c**,**g**,**h**), or 16 h (**d**–**f**,**i**). (**a**) Assembly of G-1 particles (asterisk) outside cells. (**b**) Section of a typical particle-free cell; N: nucleus, mt: mitochondria. (**d**) A small G-1 aggregate/agglomerate attached to the cell membrane (arrow). A coated pit forms in close vicinity of a G-1 particle. (**e**) Compact G-1 aggregate/agglomerate attached to a cell. (**f**) A lysosome containing electron dense G-1 material. (**f**) Energy dispersive X-ray analysis (TEM-EDX) of a G-1-containing lysosome (inset); analyzed area is marked by a white circle. Signals (in arbitrary units) were obtained for silicon (Si) and oxygen (O) at typical positions (in keV). (**g**,**h**) large aggregate/agglomerate of G-3 (asterisk) in the state of phagocytosis, and (**h**) fully internalized into a large phagosome. (**i**) Lysosomes filled with fine granular electron dense material (arrows).

To further elucidate the effects of SG we studied NR8383 cells exposed to non-bioactive G-1 (90 µg/mL, Figure 9a–f) and to bioactive G-3 (17 µg/mL) (Figure 9a–i). Despite the high concentration of G-1, loose particle agglomerates (Figure 9a,b) or compact aggregates (Figure 9d) were rarely found at the cell membrane. Cells exposed to G-1 were devoid of particle-filled lysosomes or vacuoles after 90 min (Figure 9c). After 16 h, few lysosomes contained fine granular electron dense material (Figure 9e) shown to be SiO_2_ by TEM-EDX (Figure 9f). Overall, the electron microscopic investigation of NR8383 cells exposed to G-1 revealed a very moderate uptake, possibly explaining the lack of a cytotoxic response to G-1.

As expected from the light microscopic study on G-3, cells were often found to take up large G-3 aggregates by phagocytosis (Figure 9g,h). Nevertheless, smaller particle agglomerates (<300 nm) arrived at the cell membrane as well (Figure 9g, arrow) and similar sized particles were frequently found within lysosomes (Figure 9h).

Taken together these findings confirm that under cell culture conditions, SG remained a mixture of large compact aggregates and smaller sub-micron-sized particle aggregates. It is conceivable that small particle agglomerates/aggregates are released from the micron-sized particles and that this effect may underlie the minute changes of SG following dispersion with enhanced USD energy. Furthermore, the different size classes of particles seem to be internalized by NR8383 macrophages via different routes.

#### 3.2.4. Colloidal Synthetic Amorphous Silica (CS)

##### In Vitro Test with NR8383 Macrophages

**Figure 10:**
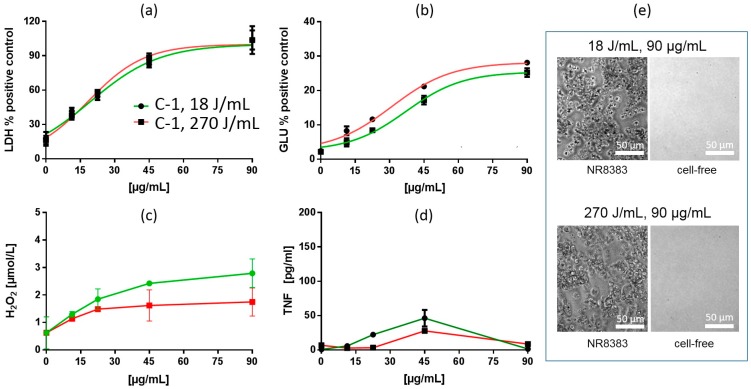
In vitro response of NR8383 alveolar macrophages to colloidal silica C-1. Particles were dispersed with either 18 J/mL (green) or 270 J/mL (red). (**a**) lactate dehydrogenase activity (LDH), (**b**) glucuronidase activity (GLU), (**c**) H_2_O_2_ concentration, and (**d**) tumor necrosis factor alpha (TNF). (**e**) NR8383 cells after 16 h exposure to P-5, dispersed with 18 J/mL or 270 J/mL; right panels show particles settled onto the bottom of culture well under cell-free conditions. Note that cells treated with both qualities appear shrunk and deteriorated; C-1 particles are not visible on phase contrast images.

Similar to the SG materials, the CS representative C-1 can hardly be disintegrated by enhanced USD energy (Table 4), as previously shown in [4]. Effects induced by C-1 treated with either 18 J/mL or 270 J/mL were highly similar, and the dose-response curves and EC50 values for LDH were nearly identical (Table 6, Figure 10a). Cytotoxic effects were also confirmed by phase contrast images (Figure 1e). However, increased USD (270 J/mL) slightly shifted the dose-response curve of GLU to the left, and diminished formation of H_2_O_2_, whereas the release of TNF remained unchanged (Figure 10).

##### Particokinetics and Interpretation of In Vitro Findings

Due to the smallness of these nanoparticles, the sedimentation velocity v_sed_, as measured for a high concentration by analytical centrifugation (Table 4) was low (0.023 mm/d). This effect was consistent for H_2_O, KRPG, F-12K (and also for degassed F-12-K at pH 8.0, data not shown) and indicates that the ionic strength typical of physiologic conditions does not precipitate C-1. In accord with this finding, phase contrast microscopy failed to show settled particles at the bottom of the culture vessel (Figure 10e). Small aggregates/agglomerates were, however, detectable by PTA in H_2_O or F-12K (Table 5). Upon increased USD energy, these particles slightly declined in size (median value) from 95 ± 15.4 to 78.8 ± 5.6 nm (after 16 h in F-12K medium), and it may be speculated that this change contributed to the minute alterations in the dose response curves of GLU and H_2_O_2_.

As previously shown, CS undergo a very slow gravitational settling from which the cellular dose at the bottom of the culture vessel can be calculated with established models, such as the ISDD model [50]. Thus, for Levasil® 200, a silica material with a size comparable to C-1 (primary particle size by TEM: 15 nm, BET: 200nm), the so-called “effective concentration” was calculated to be approximately 24.6% of the total added mass, assuming a maximum stickiness of the (cell-covered) bottom area such that particles reaching the bottom remain adhered. If it is also assumed that all these particles are taken up by the cells or will at least contact the surface, the effective concentration turns into the cellular dose. As C-1 is highly similar to the previously investigated Levasil® 200, at least with respect to size and BET, we can assume that the effective dose is close to the above reported value. A more exact estimation at the single cell level is highly desirable.

##### Electron Microscopy of Colloidal SAS

**Figure 11:**
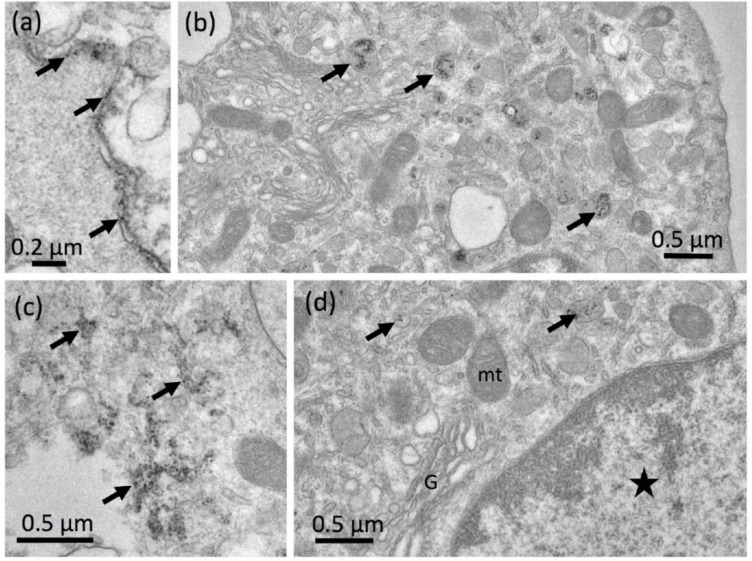
Detection of colloidal C-1 silica nanoparticle in NR8383 cells by transmission electron microscopy (TEM). Cells were treated with 22.5 µg C-1 per mL in serum-free F-12K and fixed after 90 min. (**a**) C-1 particles (arrows) located in a narrow CLEFT between two macrophages, (**b**) in electron dense lysosomes (arrows), and (**c**) in tube-like endosomal compartments (arrows). (**d**) Single nanoparticles occurred in lysosomes (arrows), but not in the cytoplasm or in organelles such as mitochondria (mt), Golgi apparatus (G), or in the nucleus (asterisk).

The subcellular distribution of C-1 was studied after 90 min of application (Figure 11a–d). As expected, we found numerous small particles that did not adhere to or destroy the cell membrane. Nevertheless, C-1 particles apparently permeated e.g., into narrow clefts formed by adjacent cells (Figure 11a), indicating their smallness under cell culture conditions. C-1 nanoparticles were frequently found in lysosomes (Figure 11b) and also in tube-like formations of the smooth endoplasmic reticulum (Figure 11c). There was no indication of the permeation of C-1 into mitochondria or cell nuclei (Figure 11d). As a whole, C-1 were taken up as small or even single nanoparticles via the endosomal route, and as they enter lysosomes and the endoplasmic reticulum.

### 3.3. General Discussion

#### 3.3.1. Errors and Exactness of Measurements

In this investigation, we analyzed the influence of two different USD energies on the biologic activity of 14 SAS by means of the alveolar macrophage assay. According to the manufacturing process, materials were grouped into PS, FS, SG and CS. This approach allowed us to identify subtle, but typical differences among materials, as evidenced by the cellular responses. Thus, administration of increased USD energy to SAS was reflected in differences in the progression of the dose-response curves registered for the release of LDH, GLU, TNF and H_2_O_2_. While statistical evaluation could be applied to LOEC values (Table 3 and Table 6), we described and interpreted logarithmically fitted slope differences and/or curve shifts for each group and on a case-by-case basis. We are aware that most of these differences are small and will hardly contribute to a differentiation between materials in a regulatory sense. However, they may be important to better understand the biological effects of SAS tested under serum-free conditions, which were chosen to uncover the cytotoxic effects of different types of SAS [54,55,56].

To reliably measure small differences, we designed the experiments in such a way that different USD energies had the largest possible impact on the outcome. To this end, all three repetitions of the macrophage assay were carried out with one stable aqueous suspension prepared from powdered material (or from the suspension “as supplied”, in the case of C-1), which was then subjected to USD energies of either 18 or 270 J/mL. Therefore, the standard deviations reported in Table 2 reflect the error caused by sample dilution and by the inevitable intra-assay variations in the biological tests, for which three successive passages of NR8383 cells were used. Furthermore, we employed a side-by-side plate design of samples treated with either 18 or 270 J/mL. All these means contributed to the comparatively small variance, and eventually allowed us to detect minute changes in curve progression, at least for LDH and GLU. However, values obtained for TNF were prone to larger standard deviations, although medium samples were retrieved from the supernatants used for LDH and GLU measurements. The progressive accumulation of TNF in the supernatant is an active cellular process, which is counteracted by cytotoxic effects of high SAS concentrations, as evidenced by biphasic dose-response curves. Furthermore, TNF detection demands a specific ELISA, which adds its own intra-assay variation to the analysis. Due to these circumstances, and especially to the biphasic responses, we were unable to apply meaningful fitting curves for TNF, and therefore, had to rely on LOECs and maximum values. Measurement of H_2_O_2_ exhibited an even larger degree of variance. As the production of H_2_O_2_ by SAS was low, at least compared to stimulation of NR8383 macrophages with lipopolysaccharide, the signal-to-noise ratio of these measurements was small. Moreover, the 90 min interval makes measurements highly sensitive to inhomogeneous settling of the particles. Therefore, we highlighted and discussed trends and mean values rather than significant differences.

#### 3.3.2. Mechanisms of SAS Toxicity in Relation to Physico-Chemical Characterization

In order to find a more general explanation for the different cytotoxic effects of all materials, we correlated physico-chemical data from Table 1 to LDH EC50 values (Table 1). Assuming that the primary particle size (by TEM), the BET surface area (by N_2_ adsorption), the number of silanol groups (by SEARS number) and/or the porosity (by uptake of DOA) might play a role in cytotoxicity, we calculated R^2^ values for the curves shown in Figure S2. No correlation with cytotoxicity was found for either primary particle size by TEM (R^2^ = 0.32), BET surface (R^2^ = 0.3), or SEARS numbers (R^2^ = 0.13). Interestingly, a weak correlation with LDH EC50 was found for porosity (uptake of DOA), which also increased from R^2^ = 0.53 to R^2^ = 0.69, if SAS with very large surface sizes (≥390 m^2^/g) such as P-2, F-3 and G-1 were excluded. With the latter restriction, BET also correlated with LDH EC50 (R^2^ = 0.51). In addition, the BET surface area was correlated with DOA uptake (R^2^ = 0.67; without P-2, F-3, and G-1: R^2^ = 0.73).

These analyses suggest that reactive SiO_2_ residues as measured by SEARS number play a minor role in macrophage toxicity for SAS, and this appears to be in contrast to previous analyses that highlight a role for silanol groups, at least for crystalline silica [57,58]. Also, the BET surface area binding surface size as such can hardly be used to predict cytotoxicity for the multitude of SAS, unless the comparison is made for closely related materials from the same production process, as shown for colloidal SAS [50], or those with very small BET values such as P-5 and F-1 (see Table 3 and Table 6). Nevertheless, the cytotoxicity of SAS may involve an interaction with molecules which may be similar to DOA with respect to size and/or chemical structure. Looking at the subcellular distribution as analyzed here, we suggest that such molecules may contact SAS in endosomal compartments or even in lysosomes, whose integrity and functioning may be compromised by a non-selective or even specific effect of SAS. Given the low cytotoxicity of G-3, together with its detection in lysosomes, it may be worthwhile studying the effects of SAS on lysosomes and their constituents more closely.

#### 3.3.3. Mechanisms of SAS Toxicity in Relation to Ultrasonic Treatment

There are several publications showing that small silica nanoparticles with a larger surface are more bioactive in vitro [9,50,59,60,61,62]. This finding also applies to some in vivo studies [21,50,63]. Concerning possible mechanisms, the size of the biologically available particles’ surface has been linked to the pro-inflammatory effect demonstrated by the release of cytokines such as interleukin 1β (IL-1β) or TNF [50], to the induction of oxidative stress [64], and also to the general cytotoxicity [21,50]. Of note, these mechanisms may be seen as separate entities [65].

However, as shown for most SAS in this study, macrophages take up aggregates and agglomerates of various sizes as well as single particles via phagosomes and smaller endosomes, such that SAS particles of identical chemical composition can be found in lysosomes, phagolysosome and probably autophagosomes, a finding also shown for other types of macrophages in vivo [66]. This makes it difficult to attribute the cellular effects observed here to a single mode of entry. However, it has been demonstrated for Hela cells that surface-functionalized colloidal silica nanoparticles (size: 55 nm) accumulate in lysosomes consequent to their endocytosis via caveolae [67]. Importantly, this mode of uptake did not lead to apoptosis or necrosis. Instead, it disturbed cellular functions, including the accumulation of autophagosomes. Also in LBC3 glioblastoma cells, silica nanoparticles were found to disturb the processing of autophagosomes, such that markers of autophagosomes like LC3-II became highly abundant [68]. Recently mechanisms that disturb autophagy have become particularly interesting in nanotoxicology because autophagosomes remove, e.g., inflammasome-associated NLRP3 complexes induced by nanomaterials. Compromising this system can aggravate, e.g., IL-1β-mediated injury [69]. Although most studies in this field used higher concentrations of SAS (up to 200 µg/mL), Wei and co-workers showed that the accumulation of LC3-II positive autophagosomes was not strictly dose-dependent but may occur progressively over time even at low concentrations [70]. The latter study was also the first to show that silica nanoparticles accumulate within the smooth endoplasmic reticulum (ER) and, by this, seem to induce ER-autophagy. This finding was recently confirmed by electron microscopy for hepatocytes [71], in which autophagosome accumulation involved the activation of the EIF2AK3 and ATF6 UPR pathways. Together these studies suggest that SAS nanomaterials have a similar mode of action on different cell types via the lysosomal pathway.

At present, harmful effects of SAS caused via uptake into lysosomes have not been shown for macrophage-like or even NR8383 cells, although a silica-mediated increase of autophagosomes has been demonstrated for RAW264.7 macrophages [72]. Inhibitor studies on THP-1 macrophages have shown that silica NP are taken up via clathrin- and/or caveolin-mediated endocytosis [73], such that their transport into lysosomes is likely. Furthermore, in THP-1 and also in bone marrow derived macrophages, autophagy and NLRP3 inflammasome turnover were compromised by rare earth nanoparticles, which had entered lysosomes [69].

The aforementioned findings are of special relevance for the results of our study: Firstly, we show that several small SAS aggregates/agglomerates of P-5, F-3, G-1, G-3, and especially C-1 quite often enter lysosomes. Furthermore, P-5 (Figure 3c P-5) or F-3 (Figure 7c) were found together with cytoplasmic material in larger vacuoles which may, therefore, be interpreted as autophagosomes. Secondly, the nano-sized SAS of C-1 and F-3 occurred in partially disrupted membrane tubing, which most likely represent parts of the ER, especially as they were found close to the Golgi apparatus. Because increased USD energy led to an erosion of nano-sized particles from larger aggregates/agglomerates, albeit to a very limited extent, we propose a dichotomous uptake. Larger particles are engulfed by phagosomes which cause low toxicity, whereas smaller particles enter into lysosomes via caveolae and the endosomal route and may be more toxic. In line with this hypothesis, larger particles such as P-6 were hardly found within lysosomes and exhibited a very moderate cytotoxicity (LOACs: 45 µg/mL). In contrast, increased numbers of SAS-laden lysosomes (inflammatory effects, cytotoxicity) co-exist as different modes of action and do not necessarily build up a chain of events [65].

Considering the small changes in LDH and GLU upon a reduction of particle size, it cannot be excluded that different entry pathways and different modes of action gradually contribute to the outcome of our experiments. However, increasing the USD energy from 18 J/mL to 270 J/mL did not lower the LOEC concentrations of LDH and GLU (Table 3 and Table 6). Instead, there was an increase in LOEAC of H_2_O_2_ release noted for 11/14 samples whereas 3/14 remained unchanged. On the other hand, the LOAC of TNF release was lowered in 6/14, increased in 3/14, and unchanged in 5/14 cases. Thus, the release of H_2_O_2_ and TNF were the most variable parameters. If only H_2_O_2_ and TNF were considered, it is important to note that FS (F-1 to F-3) showed the lowest number of possible changes (1/6), whereas PS (P-1–P-7) showed the highest number (12/14) while SG (G-1 to G-3) behaved in an intermediary way (4/6). 

This overall result can be related to the aggregate size distributions of SAS and their change by increased USD energy as investigated in detail in previous papers [4,74]. According to these findings, relatively weak USD energy (≤20 J/mL) suffices to disrupt coarse FS agglomerates into relatively rigid aggregates in the sub-micrometer range, which are slightly reduced in size upon further sonication (typically less than 40% when increasing USD energy by a factor of 10, cf. also Table 4). In contrast, PS appears to be very sensitive to USD for energy densities of 1 up to ≥1000 J/mL. Weak USD of PS yields highly polydisperse suspensions, which mainly consist of coarse agglomerates >>1 µm, but which may also contain sub-micrometer aggregates. Ongoing USD coincides with de-agglomeration, and thus a steady increase in the particles <1 µm. The extent of this size reduction depends on the specific PS product (cf. Table 4; note that DLS cannot fully reflect size changes in the micrometer range). In contrast to FS and PS, SG consists of very rigid aggregates in a size range >1 µm. The specific size distribution of SG is adjusted by milling and cannot be significantly affected by dispersion in flow fields or by USD. However, USD causes surface erosion, and thus a release of sub-micrometer or even nano-sized fragments, which are measured by DLS (but excessively overweighed). Similar to PS, the rigidity of SG aggregates and their susceptibility to USD depends on the specific SG product. Therefore, the particle size distribution, which has an influence on the biologic activity of SAS, is strongly dependent on the type of SAS, i.e., the rigidity of SAS aggregates and agglomerates, and the dispersion procedure adopted.

Finally, it is important to note that results from the alveolar macrophage assay are useful not only to compare biologic effects of similar (nano)particles, but also to prepare for further in vivo testing by means of a tiered approach. According to the criteria of the alveolar macrophage assay [29], all SAS except SG materials were found to be active (a criterion derived from short term inhalation studies [13]) and increased USD energy did not alter this categorization except for one material (G-3). However, this outcome does not necessarily imply a lung toxicity of active SAS, because in vitro assays cannot account for many factors influencing the toxicity in animals, such as protein coating in the lung lining fluid. Thus, it is well known that the addition of serum proteins drastically lowers the cytotoxic effects of SAS in vitro [54,55,56]. Furthermore, the in vitro approach cannot account for organ distribution, lung clearance, and solubility properties of SAS. Importantly, administration of SAS via inhalation occurs at a low dose rate and this condition is not reflected by the alveolar macrophage assay. Instead, the conditions of in vitro testing are better reflected by intratracheal instillation, which provides a bolus administration of a particle suspension into the lung not used or predicted for regulatory purposes. In line with this, recent experiments from our group have shown that CS similar to C-1 evoked a transient inflammation in the rat lung upon intratracheal instillation, but not after short-term inhalation [50]. Therefore, the results shown here should be understood as a worst-case scenario for AM laden with SAS under protein-free conditions, but should not be interpreted as an indication of the high toxicity of nano-sized SAS.

## 4. Conclusions

In this study on seven precipitated, three fumed, three gel and one colloidal synthetic amorphous silica (SAS) nanomaterials we investigated the biological in vitro effects of SAS dispersions prepared with two different ultrasonic dispersion (USD) energies after the removal of large particles (>10 µm) by filtration. For our study, we chose the well-established alveolar macrophage assay (based on rat NR8383 cells). Although this assay has no regulatory relevance and cannot replace in vivo testing with OECD-approved inhalation experiments, it allowed us to register full-range dose response curves under protein-free conditions for mechanistic studies. Evaluation of lactate dehydrogenase (LDH), glucuronidase (GLU), tumor necrosis factor α (TNF), and H_2_O_2_ from cell culture supernatants showed only small or gradual shifts in dose-response curves for these parameters. While cytotoxicity (release of LDH and glucuronidase) was hardly affected, H_2_O_2_ release was mostly reduced and induction of TNF showed non-uniform deviations of the low adverse effect concentration. So far, the results partially substantiate the hypothesis, namely, that the in vitro responses of NR8383 cells to SAS dispersed with increased USD energy depends on the production process of SAS. While no production process dependency was found for the cytotoxicity of SAS, induction of TNF and/or H_2_O_2_ revealed a largely uniform pattern for PS (TNF increased, H_2_O_2_ decreased) and for FS (TNF and H_2_O_2_ unchanged), whereas CS and SG behaved heterogeneously. Based on dispersion and sedimentations studies, we suggest that the small changes in biological responses were primarily attributable to an increased fraction of smaller particles, followed by changes in sedimentation and uptake. Electron microscopic studies provided evidence for at least two routes of particle ingestion. Larger particles were subject to phagocytosis, while smaller particles were preferentially taken up via caveolae and enter into lysosomes, endoplasmic reticulum, and possibly also into autophagosomes. Given the wide particle size distribution of all materials, both ways of particle uptake were simultaneously active for most materials, hence, small changes in dose-response curves observed upon higher particle dispersion energy may, at least in part, rely on this dichotomy. However, the small changes in bioactivity upon increased USD energy were not uniform and seem to depend on the production process of SAS. Therefore, we suggest that administration of moderate USD energy combined with the elimination of large particles by filtering is an adequate method to prepare SAS from different production processes for in vitro testing. Nevertheless, both the production process of SAS and the dispersion protocol need some attention when toxicological studies with SAS are designed or interpreted. 

## Figures and Tables

**Table 1:** Material properties of the synthetic amorphous silica (SAS) used in the study.

Particle Name (Abbreviation)	BET ^1)^ [m^2^/g]	CTAB ^2)^ [m^2^/g]	DOA Number ^3)^ [mL/100 g]	Sears Number ^4)^ [mL/1.5 g]	pH ^5)^	Loss on Drying ^6)^ [%]	Loss on Ignition ^7)^ [%]	Zeta-Potential ^8)^ [mV]	Point of Zero Charge ^9)^ [pH]	Solubility ^10)^ [mg/L]	Primary Particle Size by TEM ^11)^ [nm]	Aggregate Size by TEM ECD ^12)^ [NM]	Particle Size ^13)^ Mean/d50 [μm]
SIPERNAT® 160 (P-1)	180	178	268	11.1	6.1	4.0	6.5	−53	1.8	112.1	12.2 ± 2.7	58.3	12.7/11.3
SIPERNAT® 50 (P-2)	460	326	285	16.3	6.3	5.9	10.7	−21	2.4	113.9	3.1 ± 0.7	59.8	53.4/43.2
Exp. Prec. 1 (P-3)	255	255	349	14.1	7.0	5.4	10.1	−37	2.3	103.0	13.2 ± 5.2	72.05	26.4/21.6
Exp. Prec. 2 (P-4)	170	141	233	13.4	6.9	6.5	10.1	−70	1.8	89.4	19.3 ± 6.3	94.8	12.3/10.5
SIPERNAT® 22 (P-5)	180	176	213	12.9	6.5	6.1	10.2	−39	2.2	109.4	10.0 ± 2.6	82.2	116.6/117.8
Exp. Prec. 3 (P-6)	40	37	92	5.0	6.7	3.8	6.9	−35	2.1	112.3	63.8 ± 29.4	211.01	10.8/7.9
ULTRASIL® 9100 (P-7)	235	201	195	12.5	6.7	6.4	10.5	−31	2.4	122.5	16.7 ± 4.0	126.1	n.d. ^14)^
AEROSIL® OX50 (F-1)	45	69	164	1.8	4.6	<0.1	0.3	−40	2.8	117.9	41.4 ± 18.3	233.7	n.d.
AEROSIL® 200 F (F-2)	210	226	294	8.0	4.2	0.4	0.7	−27	2.6	194.0	13.5 ± 2.5	161.1	n.d.
AEROSIL® 380 F (F-3)	390	300	317	14.5	4.2	0.6	1.2	−36	2.6	226.0	8.0 ± 2.7	101.9	n.d.
Silca Gel 1 (G-1)	720	170	279	-	3.6	1.5	8.0	−29	2.8	94.2	4.9 ± 2.8	27.9	8.1/7.1
Silca Gel 2 (G-2)	340	348	287	11.8	7.5	5.0	8.3	−31	2.2	107.6	n.d.	211.6	3.1/2.7
Silca Gel 3 (G-3)	295	331	84	10.6	3.6	4.1	7.3	−28	2.6	194.5	n.d.	173.4	3.7/3.6
Colloidal Silca (C-1)	200	n.d.	n.d.	n.d.	n.d.	n.d.	n.d.	−36	4.4	n.d.	15.0	n.d.	n.d.

^1)^ N_2_ adsorption measured according to ISO 9277 and [45]. ^2)^ Cetyltrimethylammonium (CTAB) bromide surface area measured according to ISO 5794-1, the standard test method for precipitated, hydrated silica. ^3)^ Absorption of diethylhexyladipate (DOA) to 12.5 g of dry powder material was measured according ISO 19246 using a critical torque of a kneader as a measure. ^4)^ Measure for the number of silanol groups on the surface of silica as revealed by titration with alkaline solution according to Sears 1956 [46]. ^5)^ Measured in 5% solution according to ISO 787-9. ^6)^ Loss on drying was determined gravimetrically after drying at 105 °C for 2 h (modified ISO 787-2). ^7)^ Dried samples were further heated to 1000 °C for 2 h and weight loss was related to the already dried samples. ^8,9)^ Zeta potential and point of zero charge were measured with an electroacoustic sensor at a solid density of 2.1g/mL, using a DT-102 instrument (Dispersion Technology, New York, USA). ^10)^ Solubility was measured according to the enhanced OECD 105 Test Guideline on solubility, developed by “SASforREACH” (the REACH Consortium of SAS Manufacturers and Importers); a mean value from ICP-OES and UV-VIS measurements is shown. ^11,12)^ Particle size measurements were carried out with a Jeol 2010F transmission electron microscope (TEM) and ITEM software (EMSIS GmbH, Münster, Germany). To describe particle and aggregate sizes, 2000 and 1000 single elements were analyzed, respectively. ^13)^ Particles between 0.04 and 2000 µm were measured by laser diffraction with a Coulter Counter (LS 230 or LS 13320) according to DIN ISO 13320-1. ^14)^ n.d.: no data.

**Table 2:** Values from in vitro tests with the alveolar macrophage model.

Particle Name	[μg/mL]	LDH [% of pos. Control]		GLU [% of pos. Control]		H_2_O_2_ [μmol/L]		TNF [pg/mL]	
		18 J/mL	270 J/mL	18 J/mL	270 J/mL	18 J/mL	270 J/mL	18 J/mL	270 J/mL
SIPERNAT® 160 (P-1)	0	17.72 ± 4.48	17.72 ± 4.48	3.02 ± 0.90	3.02 ± 0.90	0.93 ± 0.09	0.93 ± 0.09	2.72 ± 4.21	2.72 ± 4.21
	11.25	31.34 ± 2.28	23.47 ± 2.78	7.19 ± 0.44	4.12 ± 0.33	1.17 ± 0.15	0.94 ± 0.12	8.56 ± 0.93	2.52 ± 0.64
	22.5	70.52 ± 1.98	85.58 ± 3.11	17.93 ± 0.25	21.17 ± 0.53	1.50 ± 0.29	1.08 ± 0.11	28.85 ± 1.43	33.13 ± 1.33
	45	91.21 ± 2.77	105.54 ± 4.65	28.72 ± 1.71	28.38 ± 0.93	1.97 ± 0.51	0.94 ± 0.17	43.16 ± 1.07	74.72 ± 4.32
	90	95.36 ± 3.81	96.75 ± 4.31	25.94 ± 1.80	25.93 ± 2.38	2.38 ± 0.60	0.66 ± 0.25	13.12 ± 2.62	15.66 ± 0.38
SIPERNAT® 50 (P-2)	0	16.54 ± 2.04	16.54 ± 2.04	3.28 ± 1.31	3.28 ± 1.31	0.74 ± 0.06	0.74 ± 0.06	2.72 ± 4.21	2.72 ± 4.21
	11.25	18.96 ± 1.16	15.02 ± 1.33	3.85 ± 0.24	2.62 ± 0.14	0.48 ± 0.07	0.78 ± 0.05	3.49 ± 3.82	10.25 ± 2.80
	22.5	31.80 ± 1.16	31.04 ± 2.36	6.91 ± 0.60	5.81 ± 0.38	0.98 ± 0.18	0.59 ± 0.10	5.86 ± 6.78	30.11 ± 0.75
	45	69.15 ± 3.92	75.60 ± 6.41	15.03 ± 1.16	19.76 ± 0.70	2.01 ± 0.40	1.20 ± 0.08	24.40 ± 5.67	77.28 ± 8.36
	90	83.27 ± 0.91	91.62 ± 3.61	20.28 ± 0.70	25.84 ± 0.52	3.57 ± 1.02	1.59 ± 0.37	28.39 ± 4.54	61.15 ± 5.21
Exp. Prec. 1 (P-3)	0	16.54 ± 2.04	16.54 ± 2.04	3.28 ± 1.31	3.28 ± 1.31	0.74 ± 0.06	0.74 ± 0.06	2.72 ± 4.21	2.72 ± 4.21
	11.25	22.75 ± 3.80	16.22 ± 2.86	4.34 ± 0.12	2.85 ± 0.12	0.77 ± 0.06	0.81 ± 0.07	4.94 ± 5.16	4.79 ± 5.01
	22.5	54.37 ± 4.79	64.41 ± 5.41	9.81 ± 0.50	13.75 ± 0.82	1.04 ± 0.16	0.71 ± 0.14	6.96 ± 5.44	57.96 ± 18.52
	45	95.91 ± 3.64	98.38 ± 3.66	25.26 ± 1.38	25.82 ± 0.68	1.79 ± 0.23	0.79 ± 0.15	54.26 ± 7.59	70.88 ± 10.12
	90	104.08 ± 3.92	103.36 ± 6.20	25.29 ± 1.16	30.36 ± 1.01	1.76 ± 0.42	0.94 ± 0.18	45.52 ± 11.88	67.10 ± 5.16
Exp. Prec. 2 (P-4)	0	17.37 ± 6.04	16.47 ± 3.59	2.39 ± 0.48	2.14 ± 0.24	0.99 ± 0.09	1.00 ± 0.03	6.74 ± 2.37	0.70 ± 1.57
	11.25	32.63 ± 0.31	28.44 ± 4.82	6.59 ± 0.79	4.81 ± 0.33	0.62 ± 0.45	0.66 ± 0.36	29.69 ± 1.81	9.02 ± 4.85
	22.5	84.07 ± 1.81	97.31 ± 2.76	21.20 ± 1.07	23.92 ± 0.45	1.02 ± 0.35	0.12 ± 0.19	36.88 ± 3.07	43.96 ± 5.71
	45	107.61 ± 1.37	105.62 ± 9.60	28.15 ± 1.14	28.84 ± 1.64	1.79 ± 0.44	0.19 ± 0.04	40.17 ± 11.90	33.69 ± 3.85
	90	92.57 ± 5.06	87.72 ± 3.98	25.59 ± 1.51	26.01 ± 1.80	2.34 ± 0.54	1.20 ± 0.32	19.05 ± 5.23	3.32 ± 0.82
SIPERNAT® 22 (P-5)	0	18.38 ± 3.55	18.38 ± 3.55	2.69 ± 0.79	2.69 ± 0.79	0.66 ± 0.05	0.66 ± 0.05	1.09 ± 0.40	1.09 ± 0.40
	11.25	24.87 ± 1.35	13.67 ± 0.98	4.32 ± 0.22	3.12 ± 0.16	0.53 ± 0.08	0.68 ± 0.11	4.49 ± 0.61	6.28 ± 0.93
	22.5	56.95 ± 5.61	67.32 ± 3.37	13.22 ± 0.91	14.40 ± 0.78	0.66 ± 0.14	0.47 ± 0.11	18.69 ± 1.09	48.10 ± 0.27
	45	101.68 ± 2.58	97.86 ± 3.06	27.08 ± 0.34	27.29 ± 0.66	1.36 ± 0.18	0.43 ± 0.13	53.68 ± 1.30	95.73 ± 11.95
	90	98.00 ± 1.50	98.25 ± 4.15	26.00 ± 0.22	28.59 ± 0.69	1.89 ± 0.27	0.92 ± 0.20	43.79 ± 1.33	73.25 ± 5.33
Exp. Prec. 3 (P-6)	0	18.38 ± 3.55	18.38 ± 3.55	2.69 ± 0.79	2.69 ± 0.79	0.66 ± 0.05	0.66 ± 0.05	1.09 ± 0.40	1.09 ± 0.40
	11.25	16.39 ± 2.06	14.59 ± 2.38	3.20 ± 0.22	2.55 ± 0.16	0.79 ± 0.11	0.70 ± 0.07	0.85 ± 0.49	3.36 ± 0.97
	22.5	17.52 ± 0.69	14.55 ± 1.09	4.14 ± 0.12	3.81 ± 0.11	0.70 ± 0.16	0.86 ± 0.06	4.40 ± 2.53	0.93 ± 0.46
	45	30.58 ± 0.49	31.23 ± 3.74	6.79 ± 0.21	6.49 ± 0.78	1.01 ± 0.13	0.91 ± 0.10	9.45 ± 3.69	22.82 ± 1.25
	90	56.48 ± 4.05	50.77 ± 14.52	14.63 ± 0.83	18.98 ± 3.23	1.32 ± 0.17	1.12 ± 0.06	33.03 ± 4.61	16.82 ± 6.99
ULTRASIL® 9100 (P-7)	0	16.28 ± 2.32	16.28 ± 2.32	2.96 ± 0.51	2.96 ± 0.51	0.64 ± 0.06	0.64 ± 0.06	1.09 ± 0.40	1.09 ± 0.40
	11.25	19.06 ± 1.27	16.65 ± 2.45	3.83 ± 0.17	3.42 ± 0.25	0.77 ± 0.11	0.76 ± 0.05	0.90 ± 1.38	2.05 ± 0.12
	22.5	41.03 ± 0.47	44.56 ± 7.93	10.07 ± 0.23	12.15 ± 1.36	0.66 ± 0.25	0.88 ± 0.08	17.90 ± 0.27	5.56 ± 0.33
	45	99.27 ± 1.69	101.62 ± 4.39	29.38 ± 0.60	30.64 ± 1.83	1.43 ± 0.32	1.01 ± 0.18	67.77 ± 6.03	161.59 ± 37.26
	90	101.74 ± 1.70	104.91 ± 6.60	27.08 ± 0.78	32.55 ± 0.78	1.12 ± 0.73	1.00 ± 0.21	73.29 ± 14.88	84.35 ± 11.28
AEROSIL® OX50 (F-1)	0	17.37 ± 6.04	16.47 ± 3.59	2.39 ± 0.48	2.14 ± 0.24	0.99 ± 0.09	1.00 ± 0.03	6.74 ± 2.37	0.70 ± 1.57
	11.25	13.29 ± 2.22	13.66 ± 0.58	1.89 ± 0.28	1.67 ± 0.10	0.08 ± 0.40	0.02 ± 0.19	57.56 ± 6.18	52.81 ± 2.43
	22.5	46.20 ± 5.31	29.23 ± 4.02	8.05 ± 0.86	3.60 ± 0.61	1.18 ± 0.62	0.76 ± 0.35	107.88 ± 22.71	113.81 ± 15.79
	45	101.31 ± 2.25	95.30 ± 6.10	30.97 ± 1.04	27.03 ± 1.18	0.85 ± 0.64	0.29 ± 0.38	98.99 ± 22.55	173.36 ± 7.10
	90	99.83 ± 4.76	93.81 ± 7.83	33.16 ± 1.53	31.15 ± 3.49	0.91 ± 0.64	0.11 ± 0.27	19.95 ± 1.61	45.55 ± 1.26
AEROSIL® 200 F (F-2)	0	16.17 ± 2.56	14.48 ± 1.34	1.96 ± 0.19	2.15 ± 0.18	0.65 ± 0.10	0.65 ± 0.35	0.56 ± 1.67	1.85 ± 3.09
	11.25	69.85 ± 3.91	62.47 ± 3.93	16.16 ± 1.39	14.09 ± 0.48	0.36 ± 0.08	0.12 ± 0.39	47.99 ± 4.48	38.25 ± 3.99
	22.5	107.20 ± 3.17	102.69 ± 0.62	29.04 ± 0.97	32.28 ± 0.84	0.77 ± 0.05	0.32 ± 0.37	101.55 ± 2.42	148.70 ± 14.48
	45	102.91 ± 2.83	105.01 ± 2.99	26.21 ± 1.13	32.43 ± 0.74	1.75 ± 0.10	0.82 ± 0.15	45.86 ± 4.85	68.39 ± 0.43
	90	98.75 ± 9.23	105.50 ± 7.97	23.61 ± 1.39	28.74 ± 0.89	3.54 ± 0.18	2.63 ± 0.25	30.77 ± 0.14	33.92 ± 6.19
AEROSIL® 380 F (F-3)	0	16.17 ± 2.56	14.48 ± 1.34	1.96 ± 0.19	2.15 ± 0.18	0.65 ± 0.10	0.65 ± 0.35	0.56 ± 1.67	1.85 ± 3.09
	11.25	53.86 ± 4.19	35.54 ± 4.71	10.88 ± 0.56	9.45 ± 0.48	0.43 ± 0.13	0.62 ± 0.61	39.02 ± 5.22	33.53 ± 0.51
	22.5	90.08 ± 3.57	93.39 ± 1.87	20.88 ± 1.65	23.02 ± 0.64	0.91 ± 0.20	−0.01 ± 0.39	70.98 ± 0.36	97.19 ± 3.04
	45	103.77 ± 5.81	105.42 ± 5.13	23.32 ± 0.69	26.57 ± 0.70	3.21 ± 0.58	1.57 ± 1.38	56.25 ± 1.15	117.19 ± 2.00
	90	94.26 ± 4.74	114.29 ± 5.53	21.71 ± 0.71	24.91 ± 1.05	3.70 ± 0.23	0.90 ± 1.34	23.77 ± 8.34	3.41 ± 2.08
Silca Gel 1 (G-1)	0	17.72 ± 4.48	17.72 ± 4.48	3.02 ± 0.90	3.02 ± 0.90	0.93 ± 0.09	0.93 ± 0.09	2.72 ± 4.21	2.72 ± 4.21
	11.25	9.45 ± 1.20	11.33 ± 1.60	2.66 ± 0.18	1.61 ± 0.36	0.73 ± 0.05	0.81 ± 0.03	8.45 ± 2.05	0.04 ± 0.40
	22.5	12.83 ± 0.84	13.16 ± 1.64	2.41 ± 0.39	2.11 ± 0.35	1.08 ± 0.07	0.86 ± 0.06	7.31 ± 3.15	9.86 ± 3.27
	45	15.28 ± 0.82	14.92 ± 0.74	3.10 ± 0.30	2.90 ± 0.22	0.99 ± 0.07	0.98 ± 0.09	48.01 ± 20.13	11.38 ± 0.73
	90	15.47 ± 0.97	18.37 ± 1.92	3.40 ± 0.36	3.32 ± 0.21	1.49 ± 0.21	0.90 ± 0.13	13.72 ± 15.30	0.05 ± 0.87
Silca Gel 2 (G-2)	0	17.37 ± 6.04	16.47 ± 3.59	2.39 ± 0.48	2.14 ± 0.24	0.99 ± 0.09	1.00 ± 0.03	6.74 ± 2.37	0.70 ± 1.57
	11.25	14.58 ± 0.61	15.09 ± 1.13	2.57 ± 0.33	2.13 ± 0.13	1.08 ± 0.58	0.59 ± 0.37	3.30 ± 2.52	5.40 ± 3.50
	22.5	46.90 ± 4.29	45.49 ± 2.04	7.58 ± 0.65	5.87 ± 0.47	2.00 ± 0.62	1.09 ± 0.18	6.90 ± 8.81	16.58 ± 1.64
	45	98.36 ± 1.97	98.74 ± 4.41	22.83 ± 0.44	20.14 ± 0.34	2.58 ± 0.43	1.43 ± 0.17	55.51 ± 32.93	194.66 ± 3.91
	90	102.07 ± 3.96	101.71 ± 7.64	27.21 ± 0.84	25.83 ± 0.57	4.00 ± 0.53	1.96 ± 0.22	25.61 ± 2.66	15.08 ± 8.74
Silca Gel 3 (G-3)	0	16.17 ± 2.56	14.48 ± 1.34	1.96 ± 0.19	2.15 ± 0.18	0.65 ± 0.10	0.65 ± 0.35	0.56 ± 1.67	1.85 ± 3.09
	11.25	28.95 ± 5.99	30.99 ± 3.07	3.36 ± 0.75	3.90 ± 0.21	0.24 ± 0.07	0.13 ± 0.29	14.43 ± 5.20	18.69 ± 5.53
	22.5	86.50 ± 4.12	93.39 ± 1.35	13.21 ± 1.54	22.80 ± 1.66	0.76 ± 0.08	0.81 ± 0.48	12.61 ± 7.23	20.58 ± 7.85
	45	107.37 ± 3.98	92.33 ± 3.96	24.79 ± 0.68	25.92 ± 1.28	1.44 ± 0.13	0.41 ± 0.40	38.03 ± 11.77	26.80 ± 6.19
	90	91.04 ± 5.42	105.12 ± 7.87	24.61 ± 0.87	25.75 ± 0.85	1.28 ± 0.16	0.98 ± 0.49	7.06 ± 2.01	7.80 ± 7.24
Colloidal Silca (C-1)	0	17.37 ± 6.04	16.47 ± 3.59	2.39 ± 0.48	2.14 ± 0.24	0.99 ± 0.09	1.00 ± 0.03	0.70 ± 1.57	6.74 ± 2.37
	11.25	41.77 ± 2.82	37.78 ± 3.66	8.25 ± 1.29	4.90 ± 1.18	0.74 ± 0.60	0.55 ± 0.45	5.74 ± 1.53	2.83 ± 2.32
	22.5	54.30 ± 2.98	58.49 ± 2.05	11.61 ± 0.23	8.43 ± 0.75	0.59 ± 0.24	0.52 ± 0.15	22.19 ± 2.13	3.28 ± 2.99
	45	82.47 ± 2.60	88.31 ± 3.78	21.20 ± 0.22	17.20 ± 1.26	2.34 ± 0.63	0.85 ± 0.06	46.20 ± 12.09	27.68 ± 2.29
	90	103.84 ± 8.36	103.71 ± 12.16	28.12 ± 0.54	25.21 ± 1.27	2.72 ± 0.77	1.09 ± 0.04	1.49 ± 0.63	8.59 ± 2.09

LDH: lactate dehydrogenase, GLU: glucuronidase, H_2_O_2_: hydrogen peroxide, TNF: tumor necrosis factor α. LDH and GLU values are shown in % of the Triton X-100 treated positive controls. Levels of significance are shown below.

**Table 3:** Evaluation and curve statistics for precipitated SAS.

Particle Name (Abbreviation)	Dispersion Energy	LDH				GLU				H_2_O_2_	TNF
		EC50	Hill Slope	Goodness of Fit (R2)	LOEC [μg/mL], Level of Significance	EC50	Hill Slope	Goodness of Fit (R2)	LOEC [μg/mL], Level of Significance	LOEC [μg/mL], Level of Significance	LOEC [μg/mL], Level of Significance
Corundum	18 J/mL	-	-	-	ns	-	-	-	ns	90 (***)	ns
Quartz DQ12	18 J/mL	30.88	0.03	0.97	22.5 (***)	43.39	0.03	0.9866	22.5 (***)	90 (**)	45 (***)
SIPERNAT® 160 (P-1)	18 J/mL	16.34	0.05	0.97	11.25 (***)	19.22	0.09	0.97	11.25 (***)	22.5 (*)	22.5 (***)
	270 J/mL	15.53	0.10	0.95	22.5 (***)	19.04	0.17	0.98	22.5 (***)	ns	22.5 (***)
SIPERNAT® 50 (P-2)	18 J/mL	35.29	0.02	0.94	22.5 (***)	35.23	0.04	0.99	22.5 (***)	45 (***)	45 (**)
	270 J/mL	31.72	0.03	0.96	22.5 (***)	36.25	0.06	0.99	22.5 (**)	90 (***)	22.5 (***)
Exp. Prec. 1 (P-3)	18 J/mL	20.80	0.05	0.98	22.5 (***)	26.50	0.10	0.99	22.5 (***)	45 (***)	45 (***)
	270 J/mL	19.29	0.07	0.96	22.5 (***)	24.20	0.10	0.98	22.5 (***)	ns	22.5 (***)
Exp. Prec. 2 (P-4)	18 J/mL	14.12	0.07	0.95	11.25 (***)	17.28	0.11	0.99	11.25 (***)	90 (***)	11.25 (***)
	270 J/mL	13.87	0.14	0.92	11.25 (***)	16.79	0.16	0.98	11.25 (***)	ns	22.5 (***)
SIPERNAT® 22 (P-5)	18 J/mL	19.45	0.05	0.97	22.5 (***)	23.53	0.10	1.00	22.5 (***)	45 (**)	22.5 (*)
	270 J/mL	19.04	0.08	0.95	22.5 (***)	22.93	0.14	1.00	22.5 (***)	ns	22.5 (***)
Exp. Prec. 3 (P-6)	18 J/mL	80.02	0.01	0.95	45 (***)	56.74	0.03	0.99	45 (***)	90 (**)	90 (***)
	270 J/mL	88.32	0.01	0.80	45 (***)	57.11	0.04	0.96	45 (***)	ns	45 (**)
ULTRASIL® 9100 (P-7)	18 J/mL	23.99	0.05	0.97	22.5 (***)	25.32	0.15	0.99	22.5 (***)	45 (***)	45 (***)
	270 J/mL	23.25	0.05	0.96	22.5 (***)	26.03	0.10	0.99	22.5 (***)	ns	22.5 (*)

LDH: lactate dehydrogenase, GLU: glucuronidase, H_2_O_2_: hydrogen peroxide, TNF: tumor necrosis factor α. EC50: mean effective concentration in µg/mL. LOEC: low adverse effect concentration, n.s.: not significant. Level of significance is shown in brackets with *: *p* < 0.05, **: *p* < 0.01, and ***: *p* < 0.001.

**Table 4:** Sedimentation, size and polydispersity index of selected SAS in H_2_O and F-12K medium.

Particle	Fluid	USD [J/mL]	Concentration [μg/mL]	V_sed_ [μm/s]	V_sed_ [mm/d]	X_cum_ [nm]	PDI -
P-1	H_2_O	18	485	0.584	50.49	720	0.55
	H_2_O	270	485	0.041	3.52	318	0.25
	F-12K	18	485	0.11	9.51	768	0.45
	F-12K	270	485	0.033	2.83	316	0.33
P-2	H_2_O	18	625	1.083	93.59	695	0.64
	H_2_O	270	625	0.68	58.73	487	0.43
	F-12K	18	625	1.041	89.96	656	0.74
	F-12K	270	625	0.535	46.19	473	0.43
F-1	H_2_O	18	985	0.0464	4.01	364	0.16
	H_2_O	270	985	0.0228	1.97	257	0.09
	F-12K	18	985	0.0495	4.27	410	0.16
	F-12K	270	985	0.0255	2.2	260	0.13
F-2	H_2_O	18	755	0.0071	0.62	234	0.10
	H_2_O	270	755	0.0033	0.28	177	0.12
	F-12K	18	755	0.0071	0.61	235	0.16
	F-12K	270	755	0.004	0.34	171	0.10
G-1	H_2_O	18	800	1.42	123.09	1512	0.89
	H_2_O	270	800	1.35	116.33	1032	0.65
	F-12K	18	800	1.03	88.8	2244	0.92
	F-12K	270	800	1.21	104.27	1004	0.76
G-2	H_2_O	18	945	0.35	29.84	805	0.48
	H_2_O	270	945	0.33	28.78	391	0.35
	F-12K	18	945	0.3	25.97	652	0.45
	F-12K	270	945	0.31	26.96	500	0.35
C-1	H_2_O	n.m.	5000	0.00024	0.021	n.m.	n.m.
	F-12K	n.m.	5000	0.00026	0.023	n.m.	n.m.

Particles were dispersed with two ultrasonic dispersion (USD) energies as described in the Method section and measured in either H_2_O or F-12K medium (Column “Fluid”) at concentrations suitable for DLS measurement (Column: “Concentration”); n.m.: not measured; v_sed_: sedimentation velocity, x_cum_: mean particle size, PDI: polydispersity index.

**Table 5:** Particle size in cell culture medium (tracking analysis).

Particle Name (Abbreviation)	Fluid	USD [J/mL]	DF ^1)^	Particle Size in [nm]				
				Mean	Mode	d10	d50	d90
SIPERNAT® 160 (P-1)	H_2_O	18	20	200.6 ± 5.2	127.2 ± 0.2	114.4 ± 1.4	174.5 ± 5.5	308.2 ± 2.7
	H_2_O	270	80	183.8 ± 4.0	135.4 ± 6.1	112.5 ± 0.6	154.0 ± 3.1	279.0 ± 14.8
	F-12K	18	60	134.0 ± 7.1	113.3 ± 3.1	78.5 ± 2.5	114.4 ± 6.3	212.1 ± 17.1
	F-12K	270	60	147.2 ± 5.9	102.6 ± 12.3	79.0 ± 1.7	116.3 ± 4.3	258.2 ± 15.7
SIPERNAT® 50 (P-2)	H_2_O	18	30	180.6 ± 5.6	137.7 ± 10.9	104.0 ± 3.2	149.0 ± 4.4	288.9 ± 19.0
	H_2_O	270	120	141.3 ± 1.5	105.0 ± 5.3	83.0 ± 0.8	118.5 ± 2.1	213.3 ± 5.3
	F-12K	18	1	190.8 ± 6.1	121.2 ± 6.6	103.4 ± 2.4	159.3 ± 2.7	325.7 ± 11.0
	F-12K	270	2	162.6 ± 6.9	113.7 ± 10.9	86.9 ± 0.4	130.4 ± 5.1	276.8 ± 20.5
Exp. Prec. 1 (P-3)	H_2_O	18	50	148.6 ± 3.6	116.1 ± 9.6	88.8 ± 1.0	126.6 ± 3.4	225.9 ± 13.9
	H_2_O	270	100	147.5 ± 3.3	97.5 ± 2.4	84.6 ± 1.4	124.3 ± 4.2	233.7 ± 13.9
	F-12K	18	1	197.8 ± 3.0	139.6 ± 14.6	101.9 ± 1.0	156.0 ± 5.0	370.9 ± 23.2
	F-12K	270	2	174.3 ± 3.4	122.9 ± 17.3	85.5 ± 4.3	144.0 ± 3.7	266.5 ± 13.9
Exp. Prec. 2 (P-4)	H_2_O	18	60	150.1 ± 3.9	118.3 ± 4.0	92.2 ± 1.2	125.9 ± 1.5	225.7 ± 9.5
	H_2_O	270	120	138.3 ± 2.2	93.3 ± 2.4	85 ± 1.0	119.7 ± 2.1	192.0 ± 6.0
	F-12K	18	24	151.0 ± 3.0	117.0 ± 10.9	89.2 ± 1.0	126.4 ± 2.7	245.5 ± 16.3
	F-12K	270	64	166.6 ± 4.1	125.7 ± 9.1	98.4 ± 2.9	137.1 ± 2.9	258.7 ± 29.0
SIPERNAT® 22 (P-5)	H_2_O	18	240	158.0 ± 1.1	115.6 ± 3.1	98.8 ± 2.4	135.5 ± 2.1	226.5 ± 1.9
	H_2_O	270	240	132.8 ± 3.2	103.5 ± 5.0	86.4 ± 2.1	115.6 ± 2.1	181.1 ± 11.8
	F-12K	18	20	104.3 ± 0.9	79.9 ± 2.6	51.0 ± 1.3	84.6 ± 1.4	164.3 ± 3.5
	F-12K	270	40	103.0 ± 1.9	73.9 ± 9.2	50.7 ± 1.5	80.9 ± 1.6	159.9 ± 6.2
Exp. Prec. 3 (P-6)	H_2_O	18	10	216.6 ± 4.7	145.6 ± 6.8	128.7 ± 3.5	184.3 ± 5.2	338.2 ± 6.0
	H_2_O	270	20	184.3 ± 4.0	138.1 ± 6.7	113.4 ± 2.3	156.4 ± 2.2	274.3 ± 21.9
	F-12K	18	20	99.5 ± 2.4	86.2 ± 15.6	59.0 ± 1.8	88.3 ± 6.7	142.1 ± 7.0
	F-12K	270	20	100.6 ± 2.8	82.0 ± 4.8	54.9 ± 1.6	86.4 ± 2.4	146.1 ± 6.2
ULTRASIL® 9100 (P-7)	H_2_O	18	50	157.1 ± 5.5	115.4 ± 3.9	95.8 ± 3.2	134.0 ± 4.1	233.9 ± 7.1
	H_2_O	270	100	137.0 ± 3.1	100.1 ± 3.7	85.1 ± 0.6	118.1 ± 2.3	196.4 ± 7.9
	F-12K	18	10	87.3 ± 1.9	70.5 ± 5.1	45.5 ± 2.3	69.5 ± 2.2	134.7 ± 14
	F-12K	270	10	101.5 ± 2.7	67.5 ± 2.0	53.7 ± 1.5	78.3 ± 0.8	144.2 ± 4.2
AEROSIL® OX50 (F-1)	H_2_O	18	100	218.5 ± 2.2	160.2 ± 6.2	138.9 ± 0.7	201.2 ± 3.1	306.0 ± 3.3
	H_2_O	270	100	166.4 ± 1.6	144.4 ± 14.5	112.2 ± 1.3	154.1 ± 2.8	220.7 ± 5.1
	F-12K	18	10	190.8 ± 0.6	150.2 ± 2.2	121.5 ± 1.8	172.8 ± 1.3	263.5 ± 2.8
	F-12K	270	10	164.4 ± 0.4	133.1 ± 9.2	109.1 ± 0.5	150.6 ± 0.5	224.2 ± 3.5
AEROSIL® 200 F (F-2)	H_2_O	18	400	154.1 ± 2.2	120.2 ± 8.7	98.0 ± 2.2	137.8 ± 3.1	205.7 ± 6.1
	H_2_O	270	800	137.1 ± 2.4	115.6 ± 4.9	88.7 ± 1.1	125.0 ± 1.7	188.1 ± 6.7
	F-12K	18	100	134.9 ± 1.7	116.1 ± 3.0	76.7 ± 2.1	118.8 ± 0.4	194.9 ± 6.3
	F-12K	270	200	116.3 ± 1.8	102.0 ± 10.6	62.5 ± 4.5	109.2 ± 2.3	164.4 ± 5.0
AEROSIL® 380 F (F-3)	H_2_O	18	400	141.0 ± 0.9	116.9 ± 11.4	89.5 ± 1.0	127.9 ± 0.3	194.7 ± 1.2
	H_2_O	270	400	123.5 ± 1.4	106.0 ± 3.7	80.8 ± 1.6	110.4 ± 0.4	167.3 ± 3.6
	F-12K	18	2	177.3 ± 1.9	152.0 ± 7.4	115.1 ± 3.3	161.2 ± 0.3	247.9 ± 6.3
	F-12K	270	4	156.4 ± 1.6	118.7 ± 3.0	101.6 ± 1.9	139.8 ± 3.7	218.9 ± 4.4
Silica Gel 1 (G-1)	H_2_O	18	5	209.7 ± 6.5	145.9 ± 9.1	130.0 ± 5.1	190.9 ± 5.5	302.6 ± 10.9
	H_2_O	270	5	185.3 ± 6.9	158.3 ± 18.2	108.9 ± 2.1	167.4 ± 8.1	278.9 ± 17.2
	F-12K	18	1	212.9 ± 13.1	175.0 ± 33.2	123.2 ± 1.2	200.6 ± 15.5	316.5 ± 42.6
	F-12K	270	1	189.6 ± 11.9	143.0 ± 9.3	114.6 ± 8.6	162.1 ± 2.0	285.8 ± 32.6
Silica Gel 2 (G-2)	H_2_O	18	20	247.9 ± 5.2	158.9 ± 17.5	133.5 ± 4.6	219.9 ± 2.3	392.0 ± 12.4
	H_2_O	270	40	196.1 ± 0.5	148.2 ± 7.7	110.4 ± 2.9	164.4 ± 3.8	310.1 ± 7.1
	F-12K	18	1	251.3 ± 3.8	155.4 ± 4.0	138.5 ± 5.4	217.3 ± 5.2	410.0 ± 2.1
	F-12K	270	2	201.1 ± 7.3	135.7 ± 6.2	113.6 ± 2.4	170.4 ± 6.3	323.4 ± 21.2
Silica Gel 3 (G-3)	H_2_O	18	80	154.1 ± 4.6	119.6 ± 7.3	81.9 ± 3.4	124.8 ± 4.4	255.7 ± 5.3
	H_2_O	270	80	139.7 ± 1.2	100.9 ± 3.3	79.8 ± 1.2	118.5 ± 2.1	200.6 ± 3.0
	F-12K	18	16	118.4 ± 3.4	104.3 ± 7.1	65.5 ± 0.9	100.5 ± 4.9	185.7 ± 3.7
	F-12K	270	32	122.2 ± 4.6	82.4 ± 6.4	67.2 ± 2.1	102.0 ± 4.4	187.8 ± 17.3
Colloidal Silica (C-1)	H_2_O	18	1	166.2 ± 11.7	105.3 ± 14	84.0 ± 6.3	145.5 ± 21.9	291.3 ± 41.4
	H_2_O	270	1	136.5 ± 3.1	119.6 ± 12.3	78.3 ± 3.9	124.5 ± 1.2	195.3 ± 7.2
	F-12K	18	1	95.5 ± 15.4	75.8 ± 10.5	18.9 ± 6.9	93.1 ± 9.4	162.5 ± 33.8
	F-12K	270	8	78.8 ± 5.6	62.7 ± 4.5	41.3 ± 14.5	60.6 ± 4.2	128.7 ± 11.6

Particles were dispersed in either H_2_O or F-12K medium (Column: “Fluid”) with two ultrasonic dispersion energies (Column: “USD”). ^1)^ dilution factor (DF) optimized for tracking analysis. Values for d10, d50, d90 describe the cumulative particle size distribution at 10%, 50% and 90% of the maximum value.

**Table 6:** Evaluation and curve statistics for fumed, gel and colloidal SAS.

Particle Name (Abbreviation)	Dispersion Energy	LDH				GLU				H_2_O_2_	TNF
		EC50	Hill Slope	Goodness of Fit (R2)	LOEC [μg/mL], Level of Significance	EC50	Hill Slope	Goodness of Fit (R2)	LOEC [μg/mL], Level of Significance	LOEC [μg/mL], Level of Significance	LOEC [μg/mL], Level of Significance
AEROSIL® OX50 (F-1)	18 J/mL	23.19	0.06	0.96	22.5 (***)	30.26	0.08	1.00	22.5 (***)	ns	11.25 (***)
	270 J/mL	27.70	0.05	0.95	22.5 (***)	36.35	0.09	0.99	45 (***)	ns	11.25 (***)
AEROSIL® 200 F (F-2)	18 J/mL	7.35	0.11	0.95	11.25 (***)	10.02	0.10	0.93	11.25 (***)	45 (***)	11.25 (***)
	270 J/mL	8.62	0.10	0.97	11.25 (***)	12.53	0.10	0.97	11.25 (***)	90 (***)	11.25 (***)
AEROSIL® 380 F (F-3)	18 J/mL	10.12	0.07	0.98	11.25 (***)	12.33	0.13	0.99	11.25 (***)	45 (***)	11.25 (***)
	270 J/mL	13.20	0.10	0.96	11.25 (***)	13.99	0.12	0.99	11.25 (***)	45 (***)	11.25 (***)
Silca Gel 1 (G-1)	18 J/mL	n.d			n.s.	n.d			n.s.	90 (*)	45 (***)
	270 J/mL	n.d			n.s.	n.d			n.s.	n.s.	n.s.
Silca Gel 2 (G-2)	18 J/mL	14.63	0.05	0.97	22.5 (***)	33.14	0.06	1.00	22.5 (***)	22.5 (*)	45 (***)
	270 J/mL	13.89	0.05	0.97	22.5 (***)	36.46	0.06	1.00	22.5 (***)	90 (*)	22.5 (*)
Silca Gel 3 (G-3)	18 J/mL	19.40	0.09	0.97	11.25 (**)	22.78	0.10	0.99	22.5 (***)	ns	45 (***)
	270 J/mL	18.31	0.11	0.95	11.25 (***)	17.68	0.17	0.99	22.5 (***)	ns	11.25 (*)
Colloidal Silca (C-1)	18 J/mL	19.40	0.03	0.97	11.25 (**)	30.92	0.03	0.98	11.25 (***)	45 (***)	22.5 (**)
	270 J/mL	18.31	0.04	0.97	11.25 (***)	37.15	0.04	0.98	11.25 (**)	ns	45 (**)

LDH: lactate dehydrogenase, GLU: glucuronidase, H_2_O_2_: hydrogen peroxide, TNF: tumor necrosis factor α. EC50: mean effective concentration in µg/mL. LOEC: low adverse effect concentration, n.d.: no data, n.s.: not significant. Level of significance is shown in brackets with *: *p* < 0.05, **: *p* < 0.01, and ***: *p* < 0.001.

## Supplementary Materials

The following are available online at http://www.mdpi.com/2079-4991/9/1/11/s1, Figure S1: Size distribution of synthetic amorphous silica particles (SAS) after a 16 h incubation period in distilled H_2_O and F-12K medium, Figure S2: Correlation of SiO_2_ nanoparticle-induced cytotoxicity of NR8383 macrophages with various surface parameters.
