# Supplementary material for: Effects of Ultrasonic Dispersion Energy on the Preparation of Amorphous SiO2 Nanomaterials for In Vitro Toxicity Testing
Source: Nanomaterials (Basel). 2018 Dec 22;9(1):11. doi: 10.3390/nano9010011 (PMC6359325; doi:10.3390/nano9010011)
Supplement: Supplementary file 1 [file nanomaterials-09-00011-s001.pdf]

Effects of Ultrasonic Dispersion Energy on in vitro Toxicity of Amorphous SiO_2 Nanomaterials

Supplementary Information

Martin Wiemann [1*], Antje Vennemann [1], Michael Stintz [2], Rodrigo R. Retamal Marín [2], Frank Babick [2], Gottlieb-Georg Lindner [3] Tobias Schuster [4], Ulrich Brinkmann [4], and Nils Krueger [$]

1 IBE R&D Institute for Lung Health gGmbH, Mendelstr. 11, D-48149 Münster, Germany;
2 Research Group Mechanical Process Engineering, Institute of Process Engineering and Environmental Technology, Technische Universität Dresden, Münchner Platz 3, D-01062 Dresden, Germany; rodrigo.retamal@tu-dresden.de, michael.stintz@tu-dresden.de; Frank.Babick@tu-dresden.de
3 Evonik Resource Efficiency GmbH, Brühler Straße 2, 50389, Wesseling, Germany; gottlieb-georg.lindner@evonik.com;
4 Evonik Resource Efficiency GmbH, Rodenbacher Chaussee 4, 63457 Hanau-Wolfgang, Germany; tobias.schuster@evonik.com, ulrich.brinkmann@evonik.com, nils.krueger@evonik.com;

* Correspondence: martin.wiemann@ibe-ms.de; Tel.: +49 251 9802340

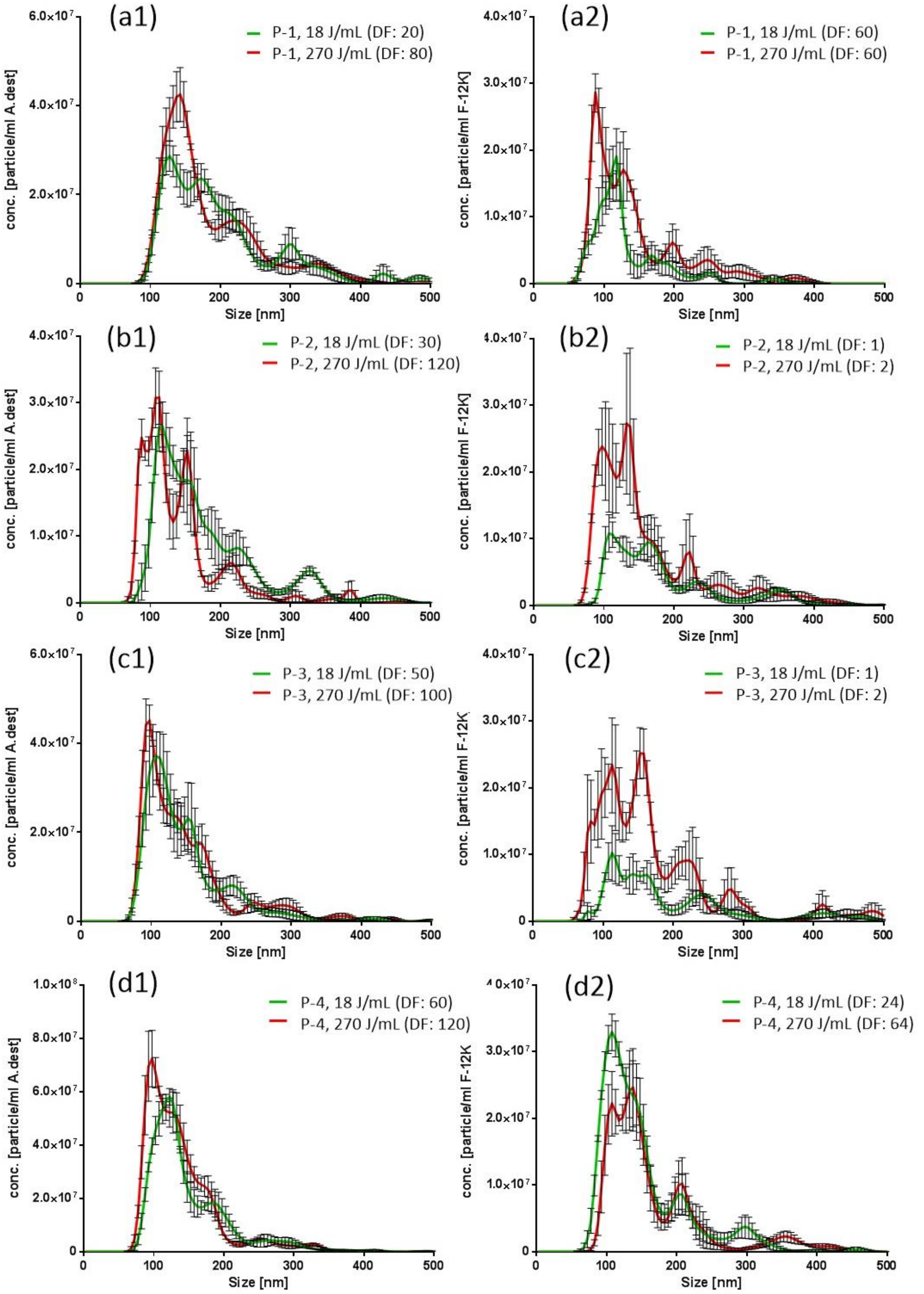

Figure S1. Size distribution of synthetic amorphous silica particles (SAS) after a 16 h incubation period in distilled H_2O and F-12K medium. Analyses were carried out by particle tracking analyses. Left diagrams (a1-n1): SAS were dispersed in distilled H_2O (A.dest), right diagrams (a2-n2): SAS were incubated in protein-free F-12K medium. Ultrasonic dispersion energy was 18 J/mL (green curves), and 270 J/mL (red curves). Curves show means and standard deviation (error bars) from three measurements. Dilution factors (DF) chosen to optimize nanoparticle tracking are given in brackets. See main paper for particle characterization and further method description.

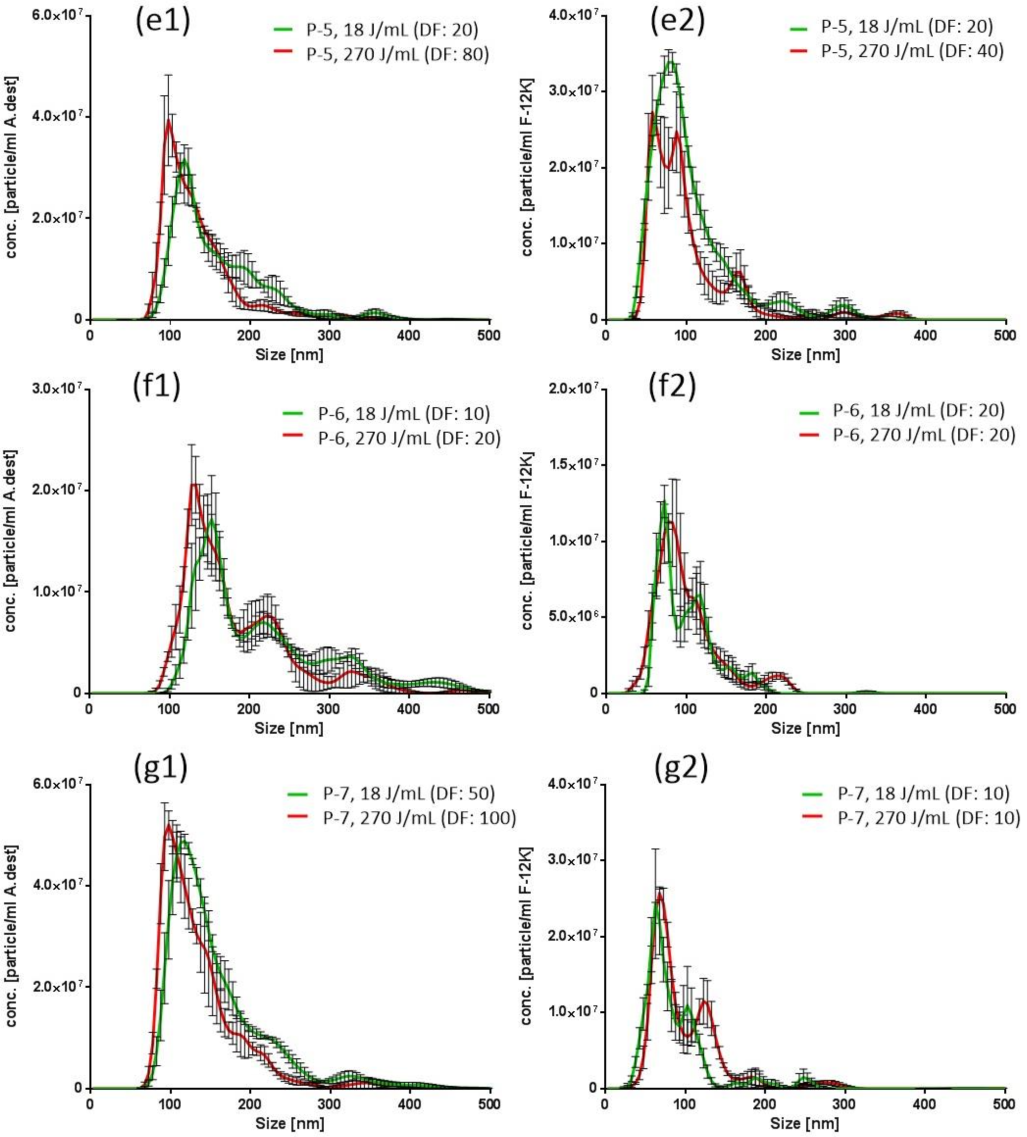

Figure S1, continued

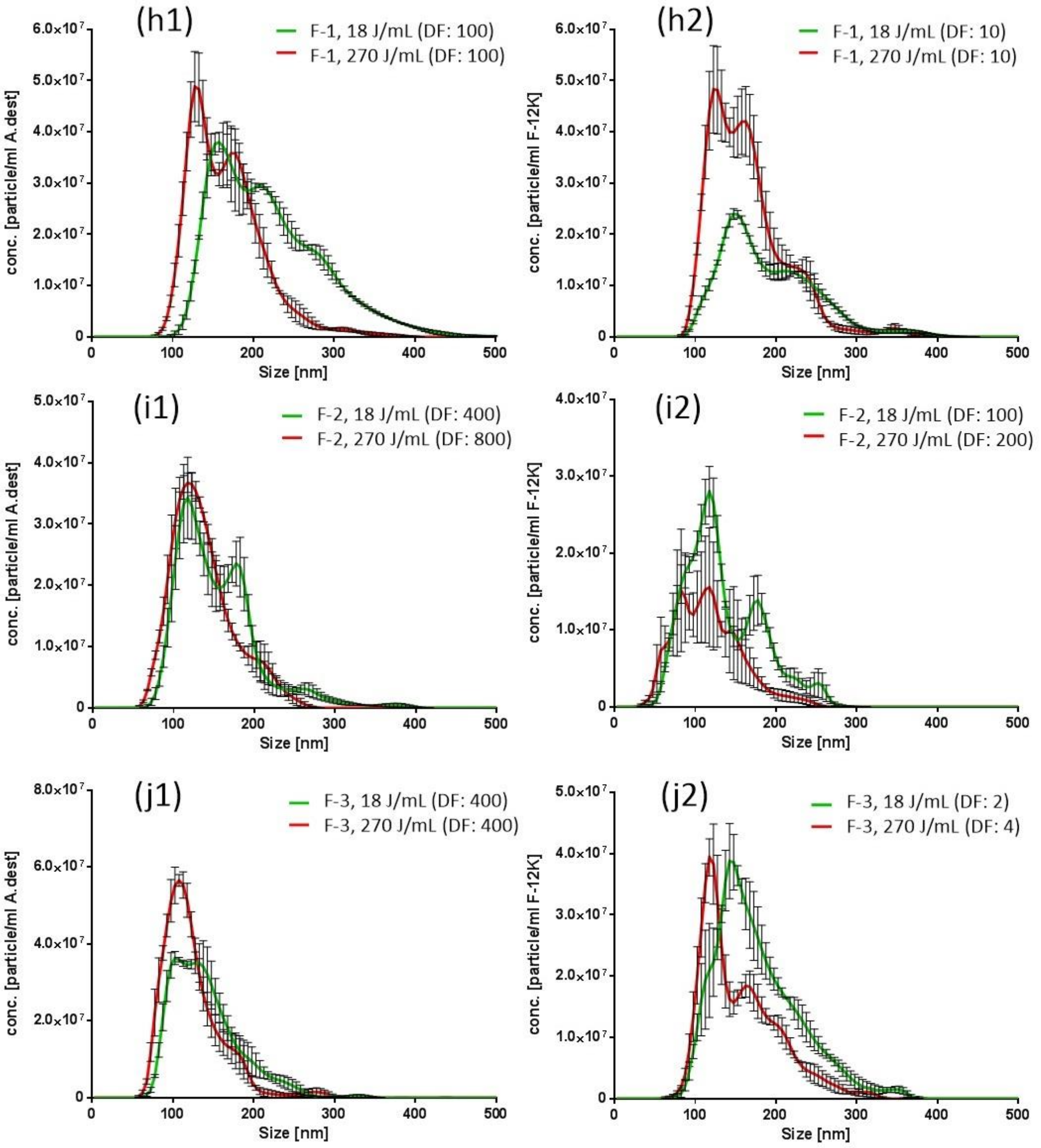

Figure S1, continued

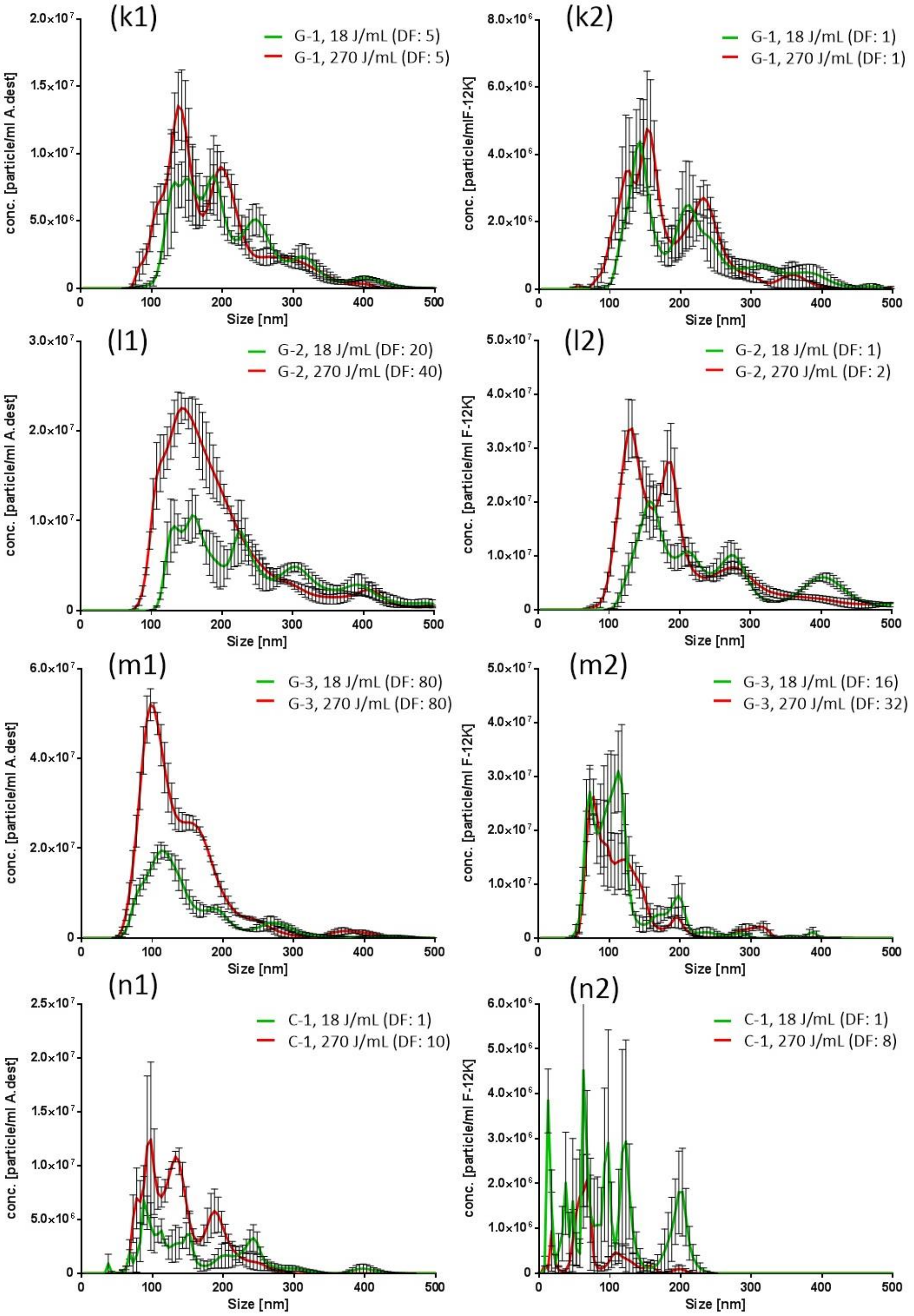

Figure S1, continued

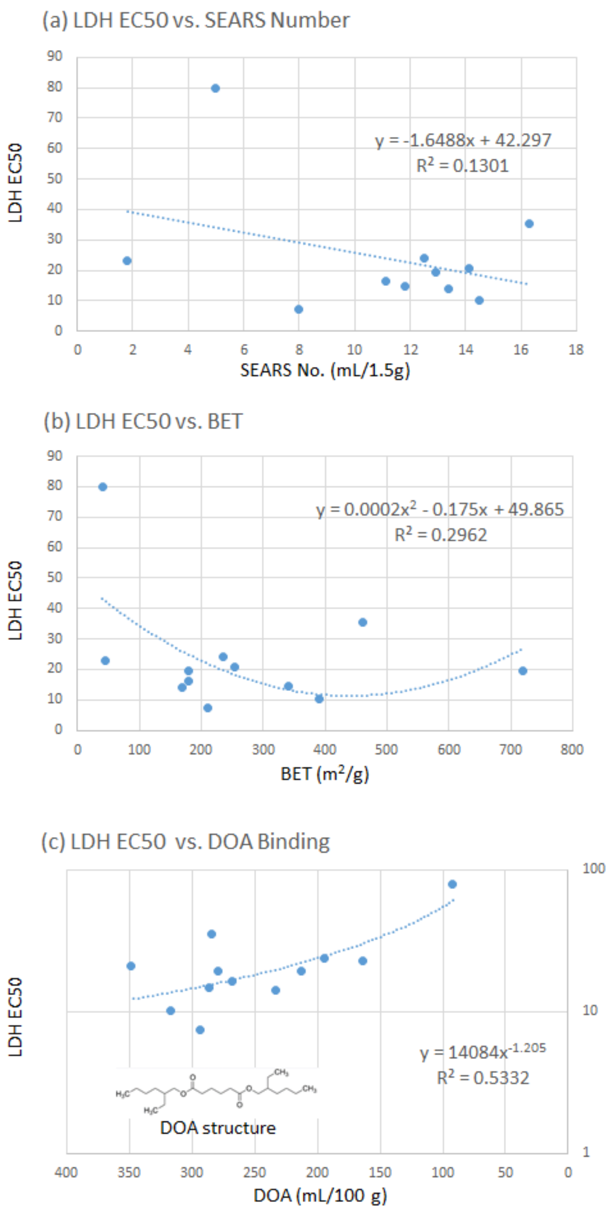

Figure S2. Correlation of SiO_2 nanoparticle-induced cytotoxicity of NR8383 macrophages with various surface parameters. Cytotoxicity is indicated by the effective concentration (EC50) for the release of LDH and was correlated with (a) Sears number, (b) N_2 binding (BET method), and (c) binding of diethylhexyladipate (DOA, see inset for formula).
